# Self‐Confined Dewetting Mechanism in Wafer‐Scale Patterning of Gold Nanoparticle Arrays with Strong Surface Lattice Resonance for Plasmonic Sensing

**DOI:** 10.1002/advs.202306239

**Published:** 2024-01-15

**Authors:** Zhiming Chen, An Cao, Dilong Liu, Zhaoting Zhu, Fan Yang, Yulong Fan, Rui Liu, Zhulin Huang, Yue Li

**Affiliations:** ^1^ Key Lab of Materials Physics Anhui Key Lab of Nanomaterials and Nanotechnology Institute of Solid State Physics HFIPS Chinese Academy of Sciences Hefei 230031 P. R. China; ^2^ University of Science and Technology of China Hefei 230026 P. R. China; ^3^ Goldots Detection technology of Hefei Co. Ltd Hefei 230000 P. R. China; ^4^ State Key Laboratory of Optical Technologies on Nano‐Fabrication and Micro‐Engineering Institute of Optics and Electronics Chinese Academy of Sciences Chengdu 610209 P. R. China

**Keywords:** non‐closely‐packed gold array, plasmonic sensing, self‐confined dewetting, surface lattice resonance, wafer scale

## Abstract

A self‐confined solid‐state dewetting mechanism is reported that can fundamentally reduce the use of sophisticated nanofabrication techniques, enabling efficient wafer‐scale patterning of non‐closely packed (*ncp*) gold nanoparticle arrays. When combined with a soft lithography process, this approach can address the reproducibility challenges associated with colloidal crystal self‐assembly, allowing for the batch fabrication of *ncp* gold arrays with consistent ordering and even optical properties. The resulting dewetted *ncp* gold nanoparticle arrays exhibit strong surface lattice resonance properties when excited in inhomogeneous environments under normal white‐light incidence. With these SLR properties, the sensitive plasmonic sensing of molecular interactions is achieved using a simple transmission setup. This study will advance the development of miniaturized and portable devices.

## Graphical abstract

A self‐confined solid‐state dewetting mechanism is proposed that can fundamentally avoid the use of sophisticated nanofabrication techniques and achieve the wafer‐scale patterning of *ncp* gold nanoparticle arrays simply and efficiently. The dewetted *ncp* gold nanoparticle arrays demonstrate non‐polarized white‐light‐excited surface lattice resonance properties in asymmetric environments, which are suitable for plasmonic sensing applications.

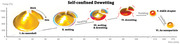

## 1 Introduction

Nanoscale gold materials have been widely used as sensitive optical transducers for biosensing and imaging owing to their intrinsic inertness and prominent surface plasmon resonance (SPR) properties, which are also categorized as plasmonic sensing.^[^ ^1^, ^2^, ^3^, ^4^, ^5^, ^6^ ^]^ The basic principle behind plasmonic sensing was established based on the plasmonic‐induced electromagnetic field confined to the gold surface, which is sensitive to dielectric changes in the surrounding medium,^[^ ^7^, ^8^, ^9^ ^]^ and where molecular interactions can be monitored in real time through a shift in their optical traits (the SPR position). This capability is pivotal for label‐free drug discovery and screening,^[^ ^10^, ^11^ ^]^ proteomic analysis,^[^ ^12^, ^13^, ^14^ ^]^ and disease diagnosis.^[^ ^15^, ^16^ ^]^ However, plasmonic resonance in metals is always accompanied by a low‐quality factor (Q‐factor) independent of the size and shape, but it is related to metal materials owing to their inherent dissipation of ohmic loss and radiative damping effect, thereby limiting detection sensitivity.^[^ ^17^, ^18^, ^19^, ^20^ ^]^ In contrast, gold nanostructures arranged in an appropriate non‐closely packed (*ncp*) periodicity can attain a unique far‐field coupling, where the localized surface plasmon resonance (LSPR) of the gold nanostructure can be coupled with one of the diffracted waves of the incident light in the plane of the array by its scattered radiation fields and propagated in a long‐range lattice mode, known as plasmonic surface lattice resonance (SLR).^[^ ^21^, ^22^, ^23^, ^24^, ^25^ ^]^ Owing to the offset of the radiative loss in each gold nanoparticle by its adjacent nanoparticles, the SLR property can achieve an ultrahigh Q‐factor (increase of approximately two orders of magnitude), offering a more promising method for plasmonic sensing.^[^ ^26^, ^27^, ^28^, ^29^, ^30^ ^]^

In practice, a 2D *ncp* gold nanostructure array with a high‐Q SLR property is more accessible and has been extensively investigated for decades, with thriving research in plasmon nanolasering,^[^ ^31^, ^32^, ^33^ ^]^ metamaterials,^[^ ^34^, ^35^ ^]^ and metaphotonics.^[^ ^36^, ^37^ ^]^ However, because of the inertness of gold, fabricating a 2D *ncp* gold array on a flat and smooth substrate primarily relies on a stenciling process in which an intermediate stencil with designed nanoholes (≈100 nm) allows the sputtering of gold to reach only the substrate surface, creating gold arrays. Moreover, the integration of stencils with other chemical methods, such as seed growth^[^ ^38^, ^39^ ^]^ or self‐assembly,^[^ ^40^, ^41^, ^42^, ^43^ ^]^ enhances the controllability of nanofabrication. However, the design of these stencils often necessitates sophisticated “top‐down” nanolithography techniques, such as electron beam lithography, which are expensive, complicated, and time consuming for achieving high resolution and centimeter scale. Moreover, exciting the SLR properties in these gold arrays from normal incidence requires a homogeneous (symmetric) environment to minimize the refractive‐index contrast between the substrate and superstrate, which is unsuitable for sensing in an asymmetric environment. Conversely, the “bottom‐up” self‐assembly method for gold nanoparticles presents a more cost‐effective alternative. For instance, Ponomareva et al. presented a simple interfacial self‐assembly approach for the fabrication of *ncp* gold nanoparticle arrays with SLR properties after introducing polymer a shell to create lattice space.^[^ ^44^ ^]^ Vogel et al. utilized colloidal lithography to produce crescent arrays that exhibit chiral surface lattice resonances.^[^ ^45^ ^]^ However, creating *ncp* spaces relies on polymer compound coatings that block further molecular binding to the gold surface.^[^ ^46^, ^47^, ^48^, ^49^ ^]^ Recently, another notable approach was the direct annealing of a thin gold nanofilm deposited on a self‐assembled colloidal crystal array to create an *ncp* gold array,^[^ ^50^, ^51^ ^]^ occupying the lattice site of the template on the substrate. However, the inherent challenges of random dislocations and defects during self‐assembly make it difficult to obtain identical colloidal crystals, resulting in poor reproducibility of SLR properties. The underlying mechanism that drives spatially oriented dewetting to form well‐ordered gold nanoparticles on flat substrates remains elusive. Therefore, bridging the high Q‐factor of SLR with plasmonic sensing remains challenging.

By scrutinizing the special thickness distribution of gold nanofilms deposited on a colloidal crystal surface, we revealed a self‐confined solid‐state dewetting mechanism that can accomplish the most intricate aspect of *ncp* gold nanoparticle array fabrication, eliminating reliance on conventional nanofabrication techniques. When combined with soft lithography, this approach enables reproducible fabrication of *ncp* gold nanoparticle arrays with identical ordering and optical properties. Notably, these dewetted *ncp* gold nanoparticle arrays demonstrate a unique strong SLR property that is excitable in an asymmetric environment under normal incidence of white light. Using these SLR properties, we designed a molecular interaction sensor with a simple transmission configuration. This design significantly simplifies the optical setup and paves the way for a high‐throughput portable commercialization process for plasmonic sensing.

## 2 Results and Discussion

### 2.1 The Concept of Self‐Confined Solid‐State Dewetting

The solid‐state dewetting of a gold nanofilm is normally disoriented and random because of film inhomogeneity, fingering instability,^[^ ^52^ ^]^ and subsequent Rayleigh instability.^[^ ^53^, ^54^ ^]^ This situation can be overcome when a gold nanofilm is sputtered onto a spherical microbead array (SiO_2_),^[^ ^55^ ^]^ where the dewetted gold nanoparticles are exclusively positioned at the top center, demonstrating a confined dewetting process that can overcome gravity, as shown in Figure S1 (Supporting Information). This confined dewetting has also been observed in the patterning of *ncp* gold nanoparticle arrays using combustible polystyrene (PS) beads as the template. A common feature of PS and SiO_2_ bead templates is their spherical surfaces, where the deposited gold layer is capable of confined dewetting during annealing, regardless of the specific template itself.

**Figure 1:**
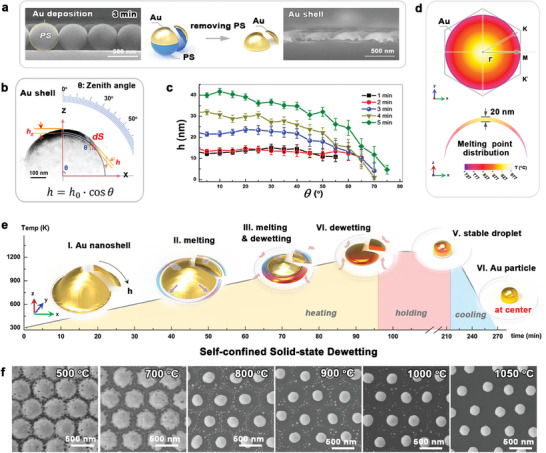
Self‐confined solid state dewetting of the gold nanoshell deposited on spherical surface. a) SEM images of gold nanoshells obtained by a gold deposition (20 mA current for 3 min) and a following removal of the spherical PS bead template by solvent. b) TEM image of a typical gold nanoshell sonicated from (a). c) The analysis of the gold nanoshell thickness relating to the measured angle and the deposition time. d) Theoretical simulation of the melting point gradient of the gold nanoshell created by its thickness. e) Schematic illustration of gold nanoshell spontaneously dewetted into a gold nanoparticle stabilized at the lattice center during the annealing treatment, defined as the self‐confined solid‐state dewetting process. f) The observation of the gold nanoshell evolving into the *ncp* array from a serial of SEM results annealed at different temperatures.

Without special instructions, we used 500 nm colloidal PS beads and gold deposition at 20 mA for 3 min in this study to reveal the dewetting mechanism during the fabrication of *ncp* gold nanoparticle arrays. **Figure** **1**a shows cross‐sectional scanning electron microscope (SEM) images of the PS bead array after gold deposition and removal of the PS template using CH_2_Cl_2_. The templating PS array reshaped the deposited gold layer into a series of gold hemi‐nanoshells. The typical transmission electron microscopy (TEM) image of the gold nanoshells shown in Figure 1b reveals a thickness gradient from the center to the side edge. This thickness variation originated from the variation in the specific surface area of the spherical beads projected onto the substrate. Because the specific surface area follows a cosine (cos) function of the Zenith angle (*θ*) rotating from the center to the measured position, the deposited gold nanoshell shows a similar variation in thickness (*h*) along the radial direction, as described in Equation (1):

(1) $$h\;=h_{0}\;\cdot \cos\theta$$

where *h_0_* is the thickness of the nanoshell at the center and *θ* is the Zenith angle. Figure 1c summarizes the thickness distribution of the gold nanoshell related to the zenith angle and gold deposition time based on the analysis of a series of TEM images shown in Figure S2 (Supporting Information). When the deposition time was too short to form consecutive gold nanoshells (Figure S2a,b, Supporting Information), the thickness distribution began validating a cosine function with the measured zenith angle until the deposition time reached 3 min. Generally, the melting point of gold decreases with decreasing gold thickness. With such radial thickness variation, the gold nanoshell attains a temperature gradient at the melting point. For instance, Figure 1d shows a theoretical simulation of the melting point distribution in a gold nanoshell (3 min gold deposition), with a 20 nm thickness at the center and a 5 nm thickness at the edge, according to the literature.^[^ ^56^, ^57^ ^]^ The gold shell demonstrates a dramatic decrease in melting point by as much as 200°C along a Γµ radial direction. This melting point gradient drives the confined dewetting of the nanoshell. Figure 1e shows a schematic illustration of the gold nanoshell annealing of gold nanoparticles. During the annealing process, the thin edge of the shell with the lowest melting temperature melted first, then dewetted and gradually receded inward onto the substrate. Once the thickest gold part melted, a liquid gold droplet formed and stabilized at the center, and further cooling produced solid gold nanoparticles. This dewetting process was directly observed in a series of SEM images of samples annealed at different temperatures, as shown in Figure 1f, proving that the dewetting orientation stems from a radial thickness difference. Because this type of confined dewetting is irrelevant to the pristine template during annealing (Figure S3, Supporting Information), we defined it as self‐confined solid‐state dewetting. Notably, an extra slight evaporation treatment at 1050°C is used to eliminate small and random gold nanoparticles, as discussed in Section S3 (Supporting Information).

### 2.2 The Influence Factors for the Self‐Confined Solid‐State Dewetting

**Figure 2:**
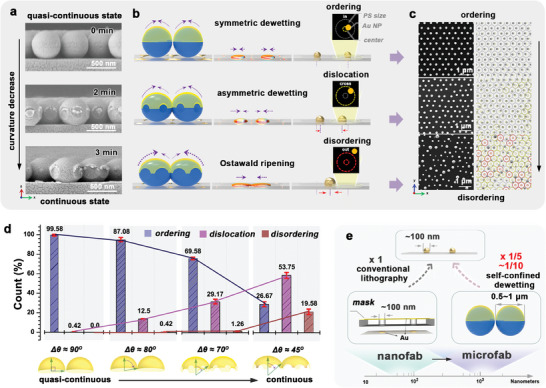
The surface curvature effect in the self‐confined dewetting. a) SEM images of gold nanofilm deposited on the colloidal crystals with a slight meltdown the templating PS bead under 110 °C for 2 and 3 min. b) Schematic illustration of three typical kinds of dewetting process and c) the corresponding SEM results. d) The relationship of the ordering of the array versus the surface curvature of the used template, based on a statistical analysis of the ordering, dislocation, and disordering percentages of the dewetted gold nanoparticles of (c). e) The self‐confined dewetting mechanism versus the conventional nanofabrication technique in the fabrication of *ncp* gold nanoparticle array.

The surface curvature of the pristine template is the primary factor influencing the self‐confined dewetting. The smaller the surface curvature, the smaller is the thickness gradient difference created in the gold nanoshell, leading to unpredictable self‐confined dewetting. **Figure** **2**a‐c demonstrates the inverse tuning of the dewetted *ncp* gold array from ordering to disordering, by reducing their surface curvature through a slight meltdown of the templating PS beads at 110°C for 1–3 min. As the surface curvature decreases, the entire array transitions from a contact‐but‐disconnected quasi‐continuous state to a connected continuous state (Figure S4, Supporting Information). Subsequent gold deposition produced gold nanoshells with smaller differences in thickness (Figure 2a). Without sufficient thickness difference to increase the melting gradient, the dewetting process became asymmetric, and the dewetted position tends to deviate from its center (Figure 2b). This asymmetric dewetting causes the dislocation of the nanoparticles to deviate from the lattice center, deteriorating the ordering of the array (Figure 2c). More severely, if the dewetting position deviates sufficiently far from its lattice center, adjacent dewetted nanoparticles can approach and merge during annealing, a phenomenon known as Ostwald ripening (third row of Figure 2a,c), forming a much larger gold nanoparticle that severely exacerbates the disordering of the array. We categorized the dewetted gold nanoparticles into ordering, dislocation, and disordering, which are marked with gray, yellow, or red dashed circles (right column in Figure 2c) to evaluate the degree of order in the *ncp* arrays (left column in Figure 2c). This classification is based on whether the dewetted position is inside, crossing, or outside the dashed circle of the spherical bead array projected onto the substrate (Figure S5, Supporting Information). In addition, owing to a constant chord length (equal to the array periodicity) on these surfaces, the varying surface curvature can be directly assessed by a cutoff Zenith angle (*θ*) along the ΓΜ radial direction, as shown in Figure S5a (Supporting Information), and labeled as *Δθ*. The valid *Δθ* value for practical gold deposition varies from 90 to 0°, where *Δθ* = 90° represents a hemispherical surface and *Δθ* = 0° represents a flat surface. Figure 2d shows the relationship between the surface curvature of the template and order of the corresponding *ncp* gold array. A slight decrease in *Δθ* from 90° to 80° dramatically increases the dislocation percentage from 0.42% to 12.5% (Figure 2d). A further decrease to 45° severely destroyed the lattice ordering of the *ncp* array, with the dislocation percentage increasing to 53.75%, and the disordering percentage increased to 19.58%. All the results prove that the surface curvature of the template is a key factor in determining the self‐confined dewetting. In addition, the arrangement of the deposited gold nanoshells is another considerable factor that influences self‐confined dewetting, and more details are discussed in Section S4 (Supporting Information).

The closely packed PS array provides a natural periodic hemispherical platform to reshape the deposited gold thickness from the center to the edge and spontaneously disconnects the gold nanoshell at the edge (where *θ =* 90°, *h* = 0), according to Equation (1). Moreover, the self‐confined dewetting mechanism changed the basic logic for fabricating the *ncp* array, as shown schematically in Figure 2e. Conventional logic relies on sophisticated nanofabrication to design an intermediate stacking mask with periodic nanoholes comparable to nanoparticle size (≈100 nm), as schematically illustrated on the left side of Figure 2e. The self‐confined dewetting mechanism requires only a periodic hemispherical surface, which pertains to the microfabrication range. As fabrication broadens from the nano‐ to microscale, the self‐confined dewetting mechanism has several fundamental advantages such as simplicity, low cost, efficiency, scalability, and suitability for most chemistry laboratories. In particular, it can be used to overcome the poor reproducibility of self‐assembly by simply replicating a periodic hemispherical surface using a soft lithography process.

### 2.3 Reproducible Dewetting of *ncp* Gold Nanoparticle Array Using Soft Lithography

**Figure 3:**
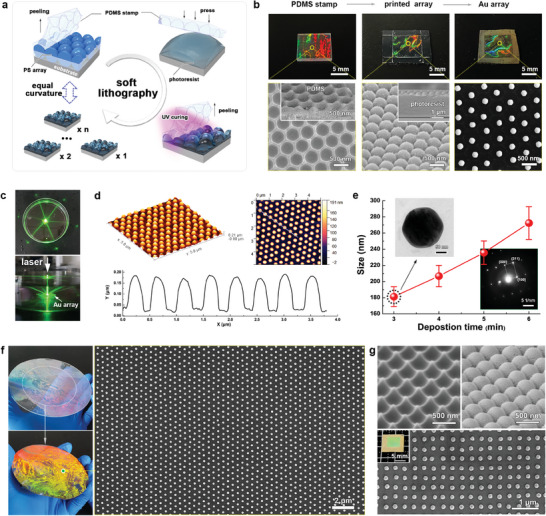
Reproducible patterning of the *ncp* gold nanoparticle arrays by soft lithography. a) Schematic illustration of the soft lithography process in constructing the photoresist arrays replicated from a colloidal crystal array. b) Typical photographs and corresponding SEM images of the PDMS stamp and the printed photoresist array involved in the soft lithography, and the final‐dewetted *ncp* gold nanoparticle array. c) A diffractive pattern of the *ncp* gold nanoparticle array in a reflective mode after a normal incidence of a green laser. d) Typical AFM results and the corresponding height measurement of the *ncp* gold nanoparticle array in (b). e) The dewetted gold nanoparticle size tunned by the gold deposition time, insets show a typical TEM image and a selected electron diffractive pattern of the gold nanoparticle obtained under 3‐min gold deposition. f) Wafer‐scale patterning of gold nanoparticle array based on soft lithography. g) SEM images of the PMDS stamp, cured photoresist array and *ncp* gold array in quadrilateral arrangement, which replicated from an AAO nanobowl template. Inset in (g) presents the photographs of the dewetted gold nanoparticle array in quadrilateral arrangement.

**Figure** **3**a illustrates the soft lithography procedure used to produce photoresist templates with a periodic hemispherical surface replicated from a self‐assembled colloidal crystal. Currently, it is effortless to obtain a well‐ordered colloidal crystal array with long‐range ordering (monocrystalline domain) over 10 cm^2^ based on an air–water interfacial self‐assembly technique, as shown in Figure S6 (Supporting Information). This monocrystalline domain was selected as the pristine template for soft lithography to guarantee high‐quality pristine ordering. Figure 3b shows typical photographs and corresponding SEM images of the soft polydimethylsiloxane (PDMS) stamp, patterned photoresist array based on soft lithography, and final *ncp* gold array. Typical photographs show interesting structural colors derived from well‐ordered arrays with 500 nm periodicity, indicating that the ordering was preserved during the soft lithography and dewetting processes. The corresponding SEM images confirm the ordering of the PDMS stamp with a hemispherical concave array and the photoresist surface patterned with a hemispherical convex array, which are complementary. Although the entire photoresist layer is up to 5 µm in thickness (as measured in Figure S7, Supporting Information), a well‐ordered *ncp* gold array has been routinely fabricated after gold deposition and annealing. These results further verified that the templating array had little effect on self‐confined dewetting after gold deposition. A laser diffraction pattern in reflective mode was used to prove the long‐range ordering of the dewetted *ncp* gold array (Figure 3c). The six clear and bright light spots indicate a large‐scale hexagonal monocrystalline lattice preserved in the gold array. Because the gold nanoparticles dewetted on the substrate had a contact angle, the diameter of the nanoparticles should be larger than their height. The AFM results are used to estimate the height of the nanoparticle, which is ≈150.5 ± 6.8 nm for 3 min gold deposition, as measured in Figure 3d. As the diameter of the gold nanoparticles is in the range of 180–200 nm (Figure 3b), the size ratio between the weight and the diameter is ≈0.75–0.83, indicating that the nanoparticles are more spherical than those directly dewetted on the substrate, as confirmed by the typical TEM image of the nanoparticle sonicated from the substrate (inset in Figure 3e). This phenomenon can be attributed to the carbonization of the polymer on the substrate, which provided a more hydrophobic contact angle. The corresponding selected‐area electronic diffraction results of the nanoparticles indicated that the gold nanoparticles were well crystallized after annealing (inset in Figure 3e). The size of the gold nanoparticles can be tuned by varying the deposition time, as shown in Figure 3e, demonstrating a nearly linear relationship. This soft lithography technique is robust and easy to scale up. Figure 3f shows the fabrication of an *ncp* gold array with an intact 4 inch scale replicated from a 6 inch wafer‐scale PDMS stamp. Moreover, the periodicity of the array can be tuned over a wide range by simply varying the size of the templated PS beads used in interfacial self‐assembly. Figure S8 (Supporting Information) shows typical dewetted *ncp* gold arrays with periodicities of 350, 750, and 1000 nm, demonstrating a well‐ordered *ncp* arrangement. Notably, the pristine template is not only restricted to a self‐assembled colloidal crystal array, but is also suitable for other templates with similar spherical surface curvatures. For example, when an anodic aluminum oxide (AAO) nanobowl array in a tetragonal arrangement was used as the pristine template (Figure S9, Supporting Information), we fabricated an *ncp* gold nanoparticle array in a tetragonal arrangement (Figure 3g; Figure S10, Supporting Information). Therefore, with the help of soft lithography, self‐confined dewetting becomes simpler, more robust, more general, more compatible, and more reproducible during the fabrication of the *ncp* gold nanoparticle arrays, which unlocks the potential for cost‐efficient commercialization.

### 2.4 The SLR Property of the Dewetted *ncp* Gold Array

**Figure 4:**
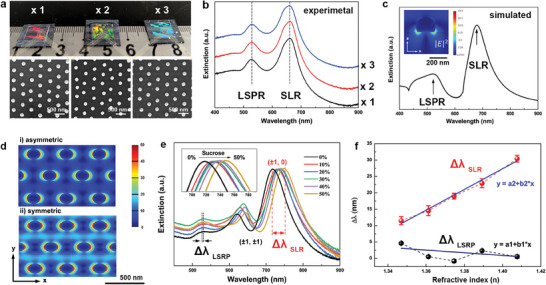
The SLR property of the dewetted *ncp* gold nanoparticle array. a) Batch fabrication of the gold nanoparticle arrays based on the same PDMS stamp printed three times. b) Experimental extinction spectra of the gold nanoparticle arrays of (a). c) FDTD simulation of the extinction spectrum and the electromagnetic field intensity of the dewetted *ncp* gold nanoparticle array of (b). d) FDTD‐calculated near‐filed distribution map of |*E/E*_0_|^2^ in asymmetric (λ = 632 nm) (i) and symmetric environments (λ = 750 nm) (ii). The period of the lattice is 500 nm, the gold nanoparticle size is 190 nm and its height is 150 nm. e) The extinction spectra in response to the change of the refractive index when immersed in a serial of sucrose solutions with the concentration varying from 0% to 50%, inset enlarges the SLR peak position. f) The wavelength shifts of the SLR and LSPR peak positions as a function of the surrounding refractive index using water as reference media. The slope a_1_ and a_2_ values of the linear fitting curves are 316 and −41 nm RIU^−1^, respectively.

Using the same PDMS stamp, it is feasible to achieve reproducible fabrication of *ncp* gold nanoparticle arrays with identical optical properties. **Figure** **4**a shows photographs and SEM images of the three dewetted *ncp* gold arrays based on photoresist array templates after repeating the soft lithography process thrice. These dewetted arrays exhibited bright but varying structural colors because of the different observation angles. The corresponding SEM results confirmed an almost identical *ncp* arrangement of the dewetted gold nanoparticles. The macro uniformity of these *ncp* gold arrays was validated by their extinction optical properties. Figure 4b shows the extinction spectra of these *ncp* gold arrays directly measured in an air/quartz asymmetric environment at a normal angle of white light incidence. They all exhibited two identical extinction peaks centered at 526 and 665 nm, indicating excellent reproducibility of soft lithography combined with self‐confined dewetting. The finite‐difference time‐domain (FDTD) algorithm was used to theoretically simulate the electric field distribution and corresponding extinction spectra of the *ncp* gold nanoparticle array, as shown in Figure 4c,d. Two extinction peaks emerged in the simulated spectrum: one centered at 523 nm was assigned to the LSPR of gold nanoparticles, and the other centered at 679 nm was the SLR of the *ncp* gold array without special dielectric matching, which is consistent with the experimental results. Compared to asymmetric environments, the SLR mode in symmetric environments is distinctly clearer, demonstrating the typical footprint of SLR (Figure 4d). According to previous studies, attaining high‐quality SLR resonance requires specific conditions including defect‐free periodic arrays, precise arrangements, and metal nanoparticles with uniform sizes and smooth surfaces.^[^ ^21^, ^22^ ^]^ Despite our efforts to optimize various experimental parameters, the dewetted arrays face challenges in maintaining high consistency in size uniformity among gold nanoparticles. Moreover, some defects persisted within the arrays during the dewetting process, such as size/shape deviations, lateral displacements, polycrystalline domains and grain boundaries of *ncp* arrays, as shown in Figure S11 (Supporting Information). These factors contributed to the relatively broad characteristic SLR peak observed in the experiment. This strong SLR property can be excited in an asymmetric environment under normal incidence,^[^ ^58^ ^]^ which is attributed to the large size of the gold nanoparticles dewetted on the substrate.^[^ ^59^ ^]^ In addition to the size effect, the spherical geometry of the dewetted gold nanoparticles can dramatically facilitate normal incident light scattering equally in all directions in the plane of the array, efficiently coupling with six adjacent nanoparticles, resulting in a stronger SLR property. Notably, the observed broad SLR observed can be readily mistaken for the quadrupole mode of large gold nanoparticles. Theoretically, the quadrupole mode of gold nanoparticles is observable in water, but challenging in air (refractive index of 1), as shown in Figure S14 (Supporting Information), which shows the spectra predicted using the Mie tool. Moreover, the presence of the SLR mode significantly reduces the strength of the quadrupole resonance, rendering it invisible in most spectra.^[^ ^60^ ^]^ Most importantly, this type of SLR resonance can fit practical sensing in an asymmetric environment, and the normal incidence angle of white light significantly simplifies the sensing optical setup, realizing an easy readout of the optical signal.

A series of proof‐of‐principle experiments was conducted to demonstrate the feasibility of white‐light‐excited SLR for plasmonic sensing. Figure S15 (Supporting Information) shows the extinction spectra of the *ncp* gold array immersed in air (reflective index, *n* = 1) and water (*n* = 1.3307). These two extinction peaks demonstrate a redshift in the wavelength, which is 7 nm for the LSPR peak and 53 nm for the SLR peak, indicating that SLR is more sensitive to changes in the surrounding refractive index than LSPR. A figure of merit (FOM* = max|[*dI(λ)*/*dn(λ)*]/*(I(λ)*|) was used to evaluate the intensity change (*dI(λ)/I(λ)*) at a fixed wavelength (*λ_o_*) in response to the refractive index change (*dn*),^[^ ^61^, ^62^ ^]^ as summarized in Figure S15b (Supporting Information). The maximum value of FOM* for the *ncp* gold nanoparticle array was ≈24, which was difficult to improve because of the broad FHWM value of the SLR. Therefore, for sensing applications, we prefer to measure the spectral shift of the SLR peak by changing the refractive index of the surrounding medium. We collected the spectra of the *ncp* gold nanoparticle array after immersion in sucrose solutions of different concentrations to vary the refractive indices, as shown in Figure 4e, to quantify the sensitivity in terms of wavelength shift per refractive index unit. The SLR peak position demonstrated a monotonic redshift in wavelength as the sucrose concentration was varied from 0% to 50%, whereas the LSRP peak position barely shifted. The appearance of the third peak is attributed to the higher‐order diffraction mode [±1, ±1] coupled with the plasmonic radiative field, as shown in Figure 4e. Figure 4f shows the relationship between the wavelength shift and refractive index changes. After linear fitting analysis, the slope for the SLR wavelength shift per refractive index unit was ≈316 nm RIU^−1^, which is much higher than that of LSPR and compatible with other excellent LSPR sensing data.^[^ ^63^ ^]^ These results indicate that white‐light‐excited SLR is suitable for use as an efficient optical transducer in plasmonic sensing.

### 2.5 SLR Sensing of the Molecular Interactions

**Figure 5:**
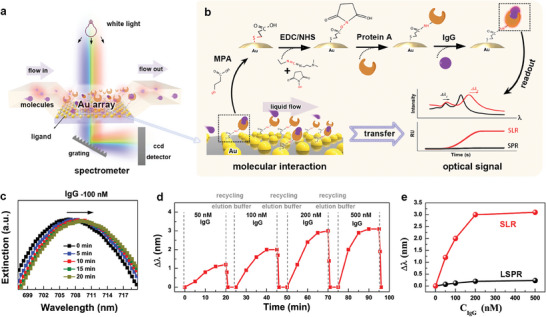
Molecular‐interaction sensing based on a *ncp* gold nanoparticle array. a) Typical working principle of the measurement. b) Schematic workflow of the surface functionalization on gold nanoparticle via EDC/NHS chemistry and the transformation of the molecular binding interaction between protein A and IgG into an easy readout of the optical signal via the *ncp* gold array. c) The dynamic evolution of the SLR peak position as the IgG molecules binding to the functional gold nanoparticle arrays over time. d) The real‐time monitoring the IgG binding at different concentrations, where the gold array chip was regenerated by elution buffer. e) The comparison of SLR and LSPR shifts in response to different concentrations of IgG binding.

With the unique features of white‐light‐excited SLR, we can design an SLR sensor in a transmission configuration that offers a more convenient equipment setup than the classical reflective Kretschamann configuration. **Figure** **5**a shows a simplified diagram of SLR sensing measurements based on the *ncp* gold nanoparticle array. The *ncp* gold array, dewetted on a quartz substrate, was sealed in a PDMS mold predesigned with a microfluidic channel that functioned as an SLR chip. A white light beam was directed perpendicularly through the gold array and collected by a spectrometer on the opposite side for analysis. Further details of the equipment setup for the SLR sensor are shown in Figure S16 (Supporting Information). Molecular binding on the gold array surface changes the surrounding refractive index, which can be measured as the extinction peak shift in the spectra. We used the SLR sensor to detect the standard molecular binding of Immunoglobulin G (IgG) to protein A, as schematically illustrated in Figure 5b, to demonstrate the sensing performance. When a solution containing the targeted IgG molecules flows through, a significant change in the refractive index around the gold array surface occurs owing to the strong specific binding between IgG and protein A, which leads to an SLR extinction peak shift. Notably, the specific molecular interaction on the gold array surface was transformed into an optical signal, allowing for real‐time readout.

Figure 5c shows the wavelength shift of the SLR peak in response to binding of 100 nm IgG molecules over time. The SLR peak exhibited a clear redshift after 20 min of association. Notably, the covalent binding of protein A molecules to the gold array also caused a redshift in wavelength, as shown in Figure S17 (Supporting Information), serving as valid evidence of successful binding. The SLR chip was regenerated by dissociating the captured IgG molecules in glycine‐HCl solution for 1–5 min, rendering it reusable for sensing applications. Figure 5d summarizes the real‐time shift in the SLR peak position in response to the association and disassociation of the IgG molecules cycled at different concentrations (50, 100, 200, and 500 nm). The SLR sensor demonstrated a gradient response to different concentrations, and additional details of the corresponding extinction spectra are shown in Figure S18, S19, Supporting Information. Figure 5e shows the response of the SLR shift versus the IgG molecule concentration, along with the LSPR peak (Figure S20, Supporting Information). The sensing performance of the SLR demonstrated a linear response in the range of 50–200 nm and then showed a saturated response at 500 nm. In comparison, the sensitivity of the SLR peak was ten times higher than that of the LSPR peak, which is in good agreement with the results shown in Figure 4e. All the sensing results indicate that SLR not only provides a promising way to refresh the sensitivity of plasmonic sensors but also enables the miniaturization of such sensors for portable detection applications.

## 3 Conclusions

We elucidated the self‐confined solid‐state dewetting mechanism of a gold nanolayer deposited on colloidal crystals. This mechanism arises from the thickness‐related melting gradient created within each gold nanoshell formed on the templating spherical surface during deposition. Self‐confined dewetting facilitates wafer‐scale fabrication of *ncp* gold nanoparticle arrays in a simple and cost‐efficient manner without requiring sophisticated nanofabrication techniques. Further combination with soft lithography overcame the reproducibility issue of the templating colloidal crystal rooted in uncontrollable self‐assembly and thus achieved batch fabrication of the *ncp* gold nanoparticle arrays with identical ordering and even optical properties. Most interestingly, these dewetted *ncp* gold arrays exhibited strong SLR properties when excited in an asymmetric environment under normal incidence of white light. This unique SLR property enables the design of a plasmonic sensor in a simple transmission configuration, which not only provides a new perspective on refreshing the sensitivity of plasmonic sensing, but also paves the way for miniaturized and portable commercialization.

## 4 Experimental Section

### Self‐Assembly of PS Colloidal Crystal Arrays

Hexagonal closely packed ordered PS colloidal crystal arrays were prepared by an air/water interface self‐assembly process. First, Si wafers or quartz glasses were cleaned with acetone/ethanol (1:1 in volume), sonicated for 15 min, rinsed with DI water, and dried under nitrogen flow. Second, the dried Si wafers and quartz glasses were treated in a hydrophilic environment using a plasma cleaner for 10 min under an oxygen atmosphere. The PS microsphere suspensions, acetone, and ethanol were then mixed in a volume ratio of 1:0.5:0.5, using ultra‐sonication. Subsequently, the PS suspension solution was dropped onto a water surface, and the PS suspension spread out, allowing the PS bead to self‐assemble at the air‐water interface, driven by the water capillary forces. As the solvent was evaporated, a monolayer of PS colloidal crystal arrays with gorgeous structural color was formed at the surface and then transferred onto the desirable substrate (Si or quartz) via a simple pick‐up process.

### Fabrication of *ncp* Gold Nanoparticle Arrays Using Soft Lithography

Briefly, a PDMS precursor with an initiator was poured into a colloidal crystal array and cured by heat. After peeling the cured PDMS, a soft PDMS stamp was obtained, with its surface pattern complementary to that of the upper surface of the colloidal array. A thin layer of the photoresist solution was spin‐coated onto the substrate and tightly compressed with the PDMS stamp for ultraviolet irradiation. When the photoresist was cured, detachment the PDMS stamp yielded a photoresist layer patterned with a hemispherical array on its surface. Several similar photoresist arrays with equal surface curvature can be produced using soft lithography. A thin layer of gold was then deposited onto the obtained photoresist array using a sputtering deposition device (Quorum, Q150RS PLUS) at 20 mA for 3 min. After annealing at 1050 °C for 2 h, an *ncp* gold nanoparticle array was obtained by self‐confined dewetting. We achieve batch fabrication of *ncp* gold nanoparticle arrays.

### The Surface Modification on the ncp Gold Nanoparticle Array

The *ncp* gold array was firstly washed with ethanol and DI water, then the clean gold array was soaked in the mercaptopropionic acid (MPA) solution (10 mm in ethanol) for 24 h at room temperature. The *ncp* gold array was rinsed thrice with ethanol and DI water to remove surplus MPA molecules, and a surface‐modified gold array was obtained after drying under nitrogen flow. The modified gold array was then sealed in a predesigned PDMS mold with a microfluidic channel and a sensor chip was prepared.

### Plasmonic Sensing of the Molecular Interaction of Protein A and IgG

The gold array surface was first functionalized with a carboxyl group (─COOH) using 3‐ MPA, which comprises a sulfhydryl group (─SH) that is strongly affinitive to the gold surface. EDC/NHS chemistry was used to activate the carboxyl groups for covalent bonding with protein‐A molecules anchored to the gold array surface as a recognition element, based on an amidation reaction between the carboxyl and amino groups of protein A. Subsequently, the IgG solution was injected into the sensor chip at an appropriate flow rate, and the extinction spectrum was recorded every 5 min. Glycine–HCl solution was used to regenerate the sensor chip. All tests were performed at room temperature.

### FDTD Simulation

Numerical simulations of the extinction spectra and electrical field distributions of the gold nanoparticle arrays were performed using the commercial software package ANSYS Lumerical FDTD 3D electromagnetic solver. All simulations were performed to match the experimental conditions. The gold nanoparticle size was set to 190 nm and the height was set to 150 nm. An infinite hexagonal lattice with a period of 500 nm was used. Periodic boundary conditions were set along both the X‐ and Y‐directions and perfectly matched layers (eight layers) were used along the *Z*‐direction. The light source was set as a plane wave with a wavelength range of 400–900 nm and was injected along the z‐axis, which had normal incidence. The refractive index of the substrate was set to 1.455 for the studied wavelength range. The permittivity of gold was modeled according to experimental data from Johnson and Christy (1972). A uniform mesh size of 2 nm was used to ensure the accuracy of the electric and magnetic field calculations for the nanoparticles.

## Conflict of Interest

The authors declare no conflict of interest.

## Supporting information

Supporting Information

## Data Availability

The data that support the findings of this study are available from the corresponding author upon reasonable request.
