# Supplementary material for: Self‐Confined Dewetting Mechanism in Wafer‐Scale Patterning of Gold Nanoparticle Arrays with Strong Surface Lattice Resonance for Plasmonic Sensing
Source: Adv Sci (Weinh). 2024 Jan 15;11(12):2306239. doi: 10.1002/advs.202306239 (PMC10966549; doi:10.1002/advs.202306239)
Supplement: Supplementary file 1 — Supporting Information [file ADVS-11-2306239-s001.pdf]

ADVANCED SCIENCE
Open Access

Supporting Information

for *Adv. Sci.*, DOI 10.1002/advs.202306239

Self-Confined Dewetting Mechanism in Wafer-Scale Patterning of Gold Nanoparticle Arrays with Strong Surface Lattice Resonance for Plasmonic Sensing

Zhiming Chen, An Cao, Dilong Liu, Zhaoting Zhu, Fan Yang, Yulong Fan, Rui Liu, Zhulin Huang and Yue Li**

Supporting Information

Self-confined Dewetting Mechanism in Wafer-scale Patterning of Gold Nanoparticle Arrays with Strong Surface Lattice Resonance for Plasmonic Sensing

Zhiming Chen,[ab#] *An Cao,*[ab#] *Dilong Liu,**[a,c] *Zhaoting Zhu,*[ab] *Fan Yang*[a], *Rui Liu,*[a] *Zhulin Huang*[a] *and Yue Li**[a]

a. Key Lab of Materials Physics, Anhui Key Lab of Nanomaterials and Nanotechnology, Institute of Solid State Physics, HFIPS, Chinese Academy of Sciences, Hefei 230031, P. R. China

b. University of Science and Technology of China, Hefei 230026, P. R. China

c. Goldots Detection technology of Hefei Co. Ltd, Hefei 230000, P.R. China

d. State Key Laboratory of Optical Technologies on Nano-Fabrication and Micro-Engineering, Institute of Optics and Electronics, Chinese Academy of Sciences, Chengdu 610209, P.R. China

[#] *These* authors contributed equally to this work.

*Address correspondence to dlliu@issp.ac.cn, yueli@issp.ac.cn

1. Experimental section

1.1 Materials

Monodisperse PS microbeads (diameter: 350 nm, 500 nm, 750 nm and 1μm) in aqueous suspensions (2.5 wt.%) were purchased from Alfa Aesar Co., Ltd. Polydimethylsiloxane (PDMS) was purchased from Dow Corning Holding Co., Ltd. Ethanol (≥ 99.7%), Acetone (≥ 99.5%), Dichloromethane (≥ 99.5%), Mercaptopropionic acid (MPA, > 99%) and Sodium dodecyl sulfate (SDS) were obtained from Sinopharm Chemical Reagent Co.,Ltd. N-Hydroxysulfosuccinimide sodium salt (NHS) and N-(3-Dimethylaminopropyl)-N′-ethylcarbodiimide hydrochloride (EDC) were purchased from Aladdin Co.,Ltd. Pig immunoglobulin G (IgG), Phosphate buffer (PBS), MES buffer and Glycine·HCl were purchased from Sangon Biotech. Protein A was obtained from Sino Biological. The target of Au (purity: 99.99%) was bought from ZhongNuo Advanced Material (Beijing) Technology Co., Ltd. All of the chemical reagents were of analytical grade and used without any purification. Deionized water (DI water) was obtained by a Milli-Q water purification system.

1.2 Self-assembly of the PS colloidal crystal arrays

The hexagonal closely-packed ordered PS colloidal crystal arrays were prepared by an air/water interface self-assembly process [1-3]. Firstly, the silicon (Si) wafers or quartz glasses were cleaned with acetone/ethanol (1: 1 in volume) and sonicated for 15 min, and then rinsed with DI water and dried with nitrogen flow. Secondly, the dried Si wafers and quartz glasses were treated hydrophilic by a plasma cleaner for 10 min under an oxygen atmosphere. Then, the PS microsphere suspensions, acetone and

ethanol were sufficiently mixed at a volume ratio of 1:0.5:0.5 via ultra-sonication. After that, the PS suspension solution was dropped into a water surface, the PS suspension will spread out, allowing the PS bead self-assembled at the air-water interface, driven by the water capillary forces. As the solvent was evaporated, a monolayer of the PS colloidal crystal arrays with gorgeous structural color was formed at the surface and then transferred onto the desirable substrate (silicon or quartz) via a simple pick-up process.

1.3 Fabrication of the *ncp* gold nanoparticle arrays via a soft lithography technique

The first step for the soft lithography technique is to obtain a PDMS stamp replicated from the PS colloidal crystal array. Briefly, a selected PS colloidal crystal on the quartz substrate was placed in a culture dish, then filled with a sufficient amount of PDMS, and cured at 75℃ for 4 h. After that, a PDMS stamp was obtained after peeled off from the quartz substrate. Similar PDMS stamps with different periodicities were prepared using PS microbeads with different diameters as the template. Then, a thin layer of optical adhesive was spun on the clean silicon substrate under a rotation speed of 1000 rpm/min, covered with the PDMS template under a force of 5 N, and allowed to stand for 5 min to ensure that all the air bubbles were completely removed. After that, the optical photoresist was cured by a UV lamp for 30 min. A well-ordered photoresist array was obtained after removing the PDMS stamp. Then, a proper thin layer of gold was deposited onto the obtained photoresist array using a sputtering deposition device (Quorum, Q150RS PLUS) at 20 mA in

current for 3 min. After annealing at 1050 ℃ for 2 h, an *ncp* gold nanoparticle array was obtained driven by the self-confined dewetting.

1.4 The surface modification on the *ncp* gold nanoparticle array

The *ncp* gold array was firstly washed with ethanol and DI water, then the clean gold array was soaked in the MPA solution (10 mM in ethanol) for 24 h at room temperature. The *ncp* gold array was rinsed with ethanol and DI water for three times to remove surplus MPA molecules, and a surface-modified gold array was obtained after drying under a nitrogen flow. The modified gold array was then sealed in a predesigned PDMS mold with a microfluidic channel, and a sensor chip was prepared.

1.5 Plasmonic sensing of the molecular interaction of protein A and IgG

The surface-modified sensor chip was incubated with EDC/NHS for activating the carboxyl groups on the surface of the Gold array. Briefly, a mixture of 20-mM EDC and 5-mM NHS (both in MES buffer) was injected into the sensor chip through a microfluidic syringe pump at a flow rate of 200 μL/min for 30 min. After activation, the excess EDC/NHS solution in the chip was washed away with PBS buffer. Then the protein A solution (50 μg/mL) was injected into the sensor chip at a flow rate of 200 μL/min for 20 min. Since the amino groups of protein A will react in an amide bond with the activated carboxyl groups on the surface of the gold array, and thus protein A was anchored onto the surface of the gold array working as the recognizing element [4]. After that, high-concentration IgG solution was diluted to 50, 100, 200 and 500 nM with PBS buffer, then injected into the sensor chip at a flow of 200 μL/min for 20 min. During that, the extinction spectrum of the gold array was recorded every 5 min. Glycine·HCl solution was injected into the sensor chip for 1

min to regenerate the sensor chip. As the IgG molecules were disassociated from the gold array surface, PBS buffer was injected into the sensor chip for 3.5 min to clean the gold array. The regenerated sensor chip was used for the next cycle of detecting the IgG molecules. All the tests were operated at room temperature.

1.6 Characterizations

The samples were characterized by field-emission scanning electron microscopy (FE-SEM, Sirion 200), transmission electron microscopy (TEM), high-resolution TEM , and energy-dispersive X-ray spectroscopy (EDS). The elemental mapping images of the products were characterized by transmission electron microscopy (FEI, Tecnai G2 F20). Samples for TEM examination were prepared by adding a droplet of the products onto a copper grid with a thin carbon film. The extinction spectra of the products were recorded on a Shimadzu 3600 spectrophotometer at room temperature. The surface of Gold arrays was characterized using atomic force microscopy (Park NX10).

2. The detailed principle of surface lattice resonance[5, 6]

2.1 Consider an array of N particles whose positions and polarizabilities are denoted $\boldsymbol{r}_i$ and α_i, respectively.

The induced dipole P_i in each particle in the presence of an applied plane wave field is

$$P_i = \alpha_i E_{loc,\,i}$$

$$(\ i = 1,2,...,N\ ) \tag{1}$$

where the local field $E_{loc,i}$ is the sum of the incident and retarded fields of the

other N-1 dipoles. At any given wavelength λ the field is equal to

$$E_{loc,i} = E_{inc,i} + E_{dipole,i} = E_0 \exp(ik \cdot r_i) - \sum_{\substack{j=1 \\ j \neq 1}}^{N} A_{ij} \cdot P_j$$

$$(i = 1,2,... N) \qquad (2)$$

where E_0 and $k = 2\pi / \lambda$ are the amplitude and wave vector of the incident wave, respectively. The dipole interaction matrix A is expressed as

$$\mathrm{A}_{ij} \cdot \mathrm{P}_j = k^2 \exp(ik \cdot r_{ij}) \frac{r_{ij} \times (r_{ij} \times P_j)}{r^3_{ij}} + \exp(ik \cdot r_{ij})(1 - ik \cdot r_{ij}) \frac{\left[r^2_{ij} P_j - 3 r_{ij} (r_{ij} \cdot P_j) \right]}{r^5_{ij}}$$

$$(i = 1,2,...,N, \quad j = 1,2,..., N, \quad j \neq i) \qquad (3)$$

where $\boldsymbol{r}_{ij}$ is the vector between dipole i and dipole j.

For an infinite NP arrays and the wavevector k is perpendicular to the NP plane, the induced polarization in each NP is the same. We can generate an analytical solution of equation (3), the polarization $\boldsymbol{P}$ and extinction cross section $\boldsymbol{C}_{\text{ext}}$ of each particle can be written as:

$$P = \frac{\partial_s E_0}{1 - \partial_s S}$$

$$C_{ext} = 4\pi N k \,\mathrm{Im}\left(\frac{P}{E_0} \right) \qquad (4)$$

where S is the retarded dipole sum,

$$S = \sum_{j \neq i} \left[\frac{(1 - ikr_{ij})(3\cos^2 \theta_{ij} - 1) e^{ikr_{ij}}}{r^3_{ij}} + \frac{k^2 \sin^2 \theta_{ij} e^{ikr_{ij}}}{r_{ij}} \right] \qquad (5)$$

where $\boldsymbol{\theta}_{\text{ij}}$ is the angle between the polarization vector (in the plane of the array) and $\boldsymbol{r}_{\text{ij}}$ is the vector from dipole i to dipole j.

The exact condition of the excitation of surface lattice resonances (SLR) is the polarization of $\boldsymbol{P}$ becomes largest, that means that the real part of $1 / \alpha_{\text{S}} - S$ equals to zero. The linewidth of SLR is governed by the imaginary part of $1 / \alpha_{\text{S}} - S$.

For small spherical particles close to resonance, the polarizability α_{S} is

$$\partial_s = -\frac{A}{(\omega - \omega_p + i\gamma)} \tag{6}$$

where *A* is a constant,

$$A = \frac{1}{2}\omega_p r^3 \tag{7}$$

And ω_p is the surface plasmon frequency for the isolated particle and γ is its half-width, by substituted this equation into Eq. (4) we find:

$$P = \frac{-AE_0}{\omega - \omega_p + i\gamma + AS} = \frac{-AE_0}{\omega\{\omega_p - \mathrm{Re}(AS)\} + i\{\gamma + \mathrm{Im}(AS)\}} \tag{8}$$

2.2 For a hexagonal lattice, the theoretical wavelengths λ_{mn} of the Bragg diffraction modes were calculated by:

$$\lambda_{mn} = RI*d \tag{9}$$

where *RI* is the refractive index and *d* is the interplanar spacing which can be calculated by:

$$\frac{1}{d^2} = \frac{4}{3}\left(\frac{m^2 + mn + n^2}{a^2}\right) \tag{10}$$

where *m* and *n* are the Miller indices (in our case ±1 and 0, respectively), and *a* is the lattice constant corresponding to the nearest-neighbor interparticle distance (in our case 500 nm)[7-9]. By substituting the periodicity and the refractive indices of the fused quartz substrate (n_d=1.455) and air (n_d=1.000) in eq 9, we obtain the λ_{mn} of 630 nm and 433 nm on the substrate side and the air side, respectively. The former can strongly couples into the plasmon modes of individual unit cells when the wavelengths of diffraction 630 nm and LSPR (~620 nm for 200 nm NPs) get close to each other, resulting in a true SLR.

3. The slight evaporation treatment during the self-confined solid-state dewetting

Since the PS beads assembled in a 2D closely-packed array are spatially contacted but disconnected, the sputtered gold nanoshells will also arrange in a contacted but disconnected state, defined as a quasi-continuous state distinguished from the continuous state and the discrete state. Besides the formation of gold nanoshells, sputtering gold can also cross the interparticle voids of the template, producing a series of triangle gold nanoplates on the substrate. These gold nanoplates are discrete and spatially separated from the gold nanoshells, and usually have little effect on the self-confined dewetting process but have to be removed. Moreover, the relative surface area ratio between the gold nanoplates and the gold nanoshell is very low (around 7%), indicating that the nanoparticle dewetted on the lattice site should be much larger than that of the void site (Figure S21). Theoretically, their nanoparticle volume ratio is approaching 13.9, making it feasible to eliminate the void-sited nanoparticles through a slight evaporation treatment.

Scheme S1 illustrates the dewetting fabrication of the ncp gold nanoparticle array based on using a closely-packed PS bead array as the template. At the beginning of the annealing, the PS template will melt and burn off at a relatively low temperature (< 450 ^{o}C), exposing the spherical gold nanoshells and the triangle gold nanoplates on the substrate. Then the gold nanoshells tend to dewet into large gold nanodroplets occupying the lattice sites, driven by the self-confined dewetting. In contrast, driven by the solid-state dewetting, the gold nanoplates prefer to dewet into several small gold nanodroplets that are randomly distributed at the void area. To remove these void-sited gold droplets, a slight evaporation treatment at 1050 ^{o}C for 2h was additionally employed, which only cause a mild decrease of the lattice-sited gold

droplets in size at the same time. Further cooling these lattice-sited gold nanodroplets produces the ncp gold nanoparticle array. More evidence results for the ncp gold nanoparticle array before and after a slight evaporation treatment were presented in Figure S22. It can be found that the void-sited gold nanoparticles (42.5 ± 12.8 nm in diameter) were eliminated after the treatment, and the lattice-sited gold nanoparticles only have a size decreases from 191.1 (± 15.4) nm to 182.3 (± 12.8) nm. Moreover, a higher temperature (such as 1100 ^{o}C) can accelerate the evaporating rate but causes an unavoidable size destruction of the lattice-sited nanoparticles in the array (Figure S23). Therefore, the ordering of the ncp array is strongly dependent on the self-confined dewetting process that rules the final dewetted position of the gold nanoparticles.

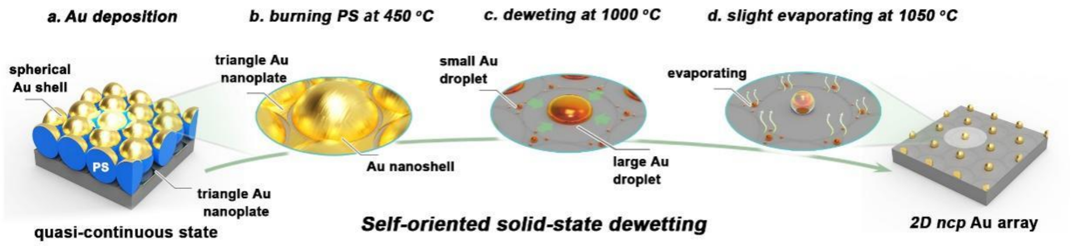

Scheme S1. Schematic illustration of the self-confined dewetting process of the gold nanofilm deposited on a colloidal crystal array of PS beads, where the deposited gold layer was reshaped into a serial of spherical nanoshells and triangle nanoplates that are spatially detached from each other, known as a quasi-continuous state.

4. A quasi-continuous arrangement of the gold nanoshell

Besides the pristine surface curvature, the arranging state of the deposited gold nanoshells is another considerable influence factor for self-confined dewetting. With the decreasing of the templating surface curvature, the deposited gold nanoshells tend to connect and gradually evolve into a continuous state (Figures S5b to S5e). In that

case, the self-confined dewetting at each site will be intertwined with its adjacent sites through their connected edges, which will intensify the position dislocation or even trigger the Ostwald ripening. For example, by prolonging the deposition time to 8 min, the gold nanoshells will arrange in a kind of partially continuous state and then cause the Ostwald ripening process, resulting in a localized disordering of the array (Figure S24). A discrete state of the gold nanoshells can avoid such adjacent interactions, but the rest interparticle void area expands and will be covered with a continuous and net-like gold nanofilm after the gold deposition, making it hard to be removed through the additional evaporation treatment during the annealing. In that case, to realize a well-ordered self-confined dewetting, it requires the deposited gold nanoshells not only with sufficient thickness gradient but also arranged in a balanced quasi-continuous state.

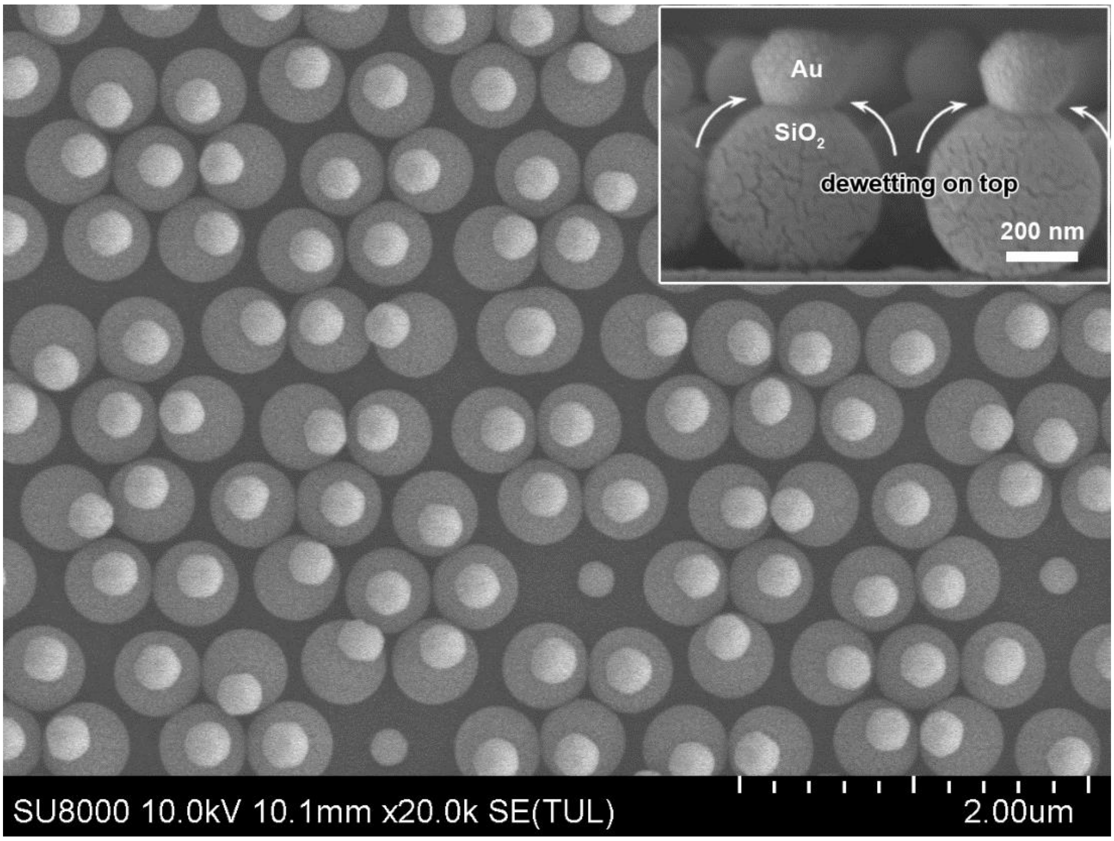

Figure S1. SEM images of gold nanoparticles preferentially dewetted onto the top of SiO_2 nanoparticles when a SiO_2 nanoparticle array was sputtered with a thin layer of gold film and annealed at 1050 ^oC for 2h.

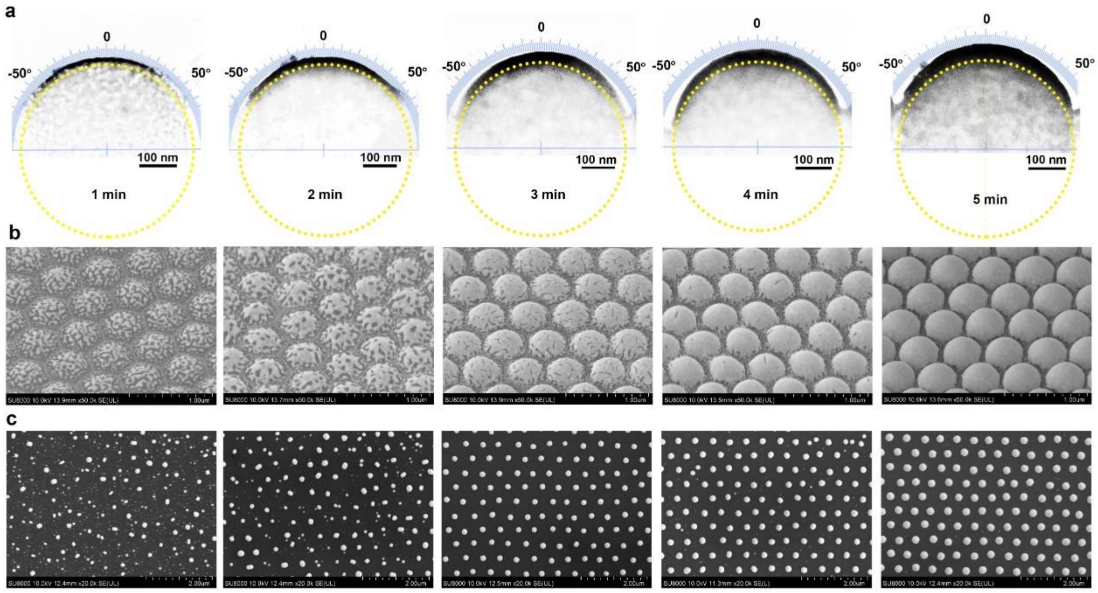

Figure S2. The thickness of gold nanoshell formed on PS bead relating to its deposition time. (a) TEM images of typical gold nanoshells with deposition time varying from 1 min to 5 min. (b) Corresponding 45-degree-tilted-view SEM images of these gold nanoshell arrays after removing the PS bead template. The gold shell evolves from a grainy rough surface into a smooth gold nanoshell, which is similar to the “snow” deposited on land, with increasing the deposition time. (c) The corresponding gold nanoparticle arrays after annealed the gold nanoshells of (b) at 1050 ^oC for 2 h.

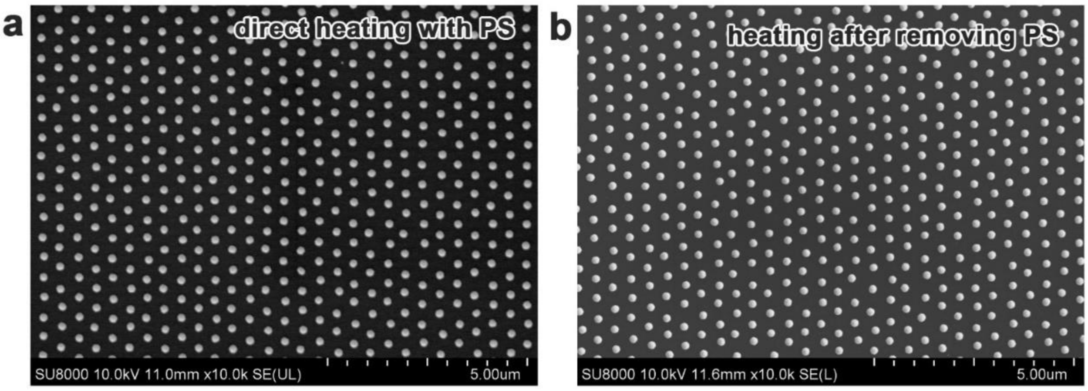

Figure S3. The comparison of the dewetted gold nanoparticle arrays formed without (a) and with (b) removing the PS bead template before the annealing process.

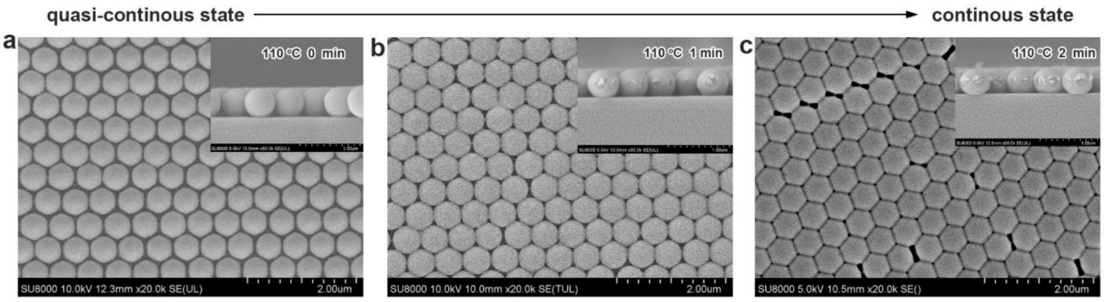

Figure S4. Typical top-view and cross-sectional-view SEM images of the PS bead array after melting at 110 ^{o}C for 0 min (a), 1 min (b), and 2 min (c). The PS bead array evolves from a quasi-continuous state to a continuous state after slightly melting the PS bead to merge under the polymer glassifying temperature.

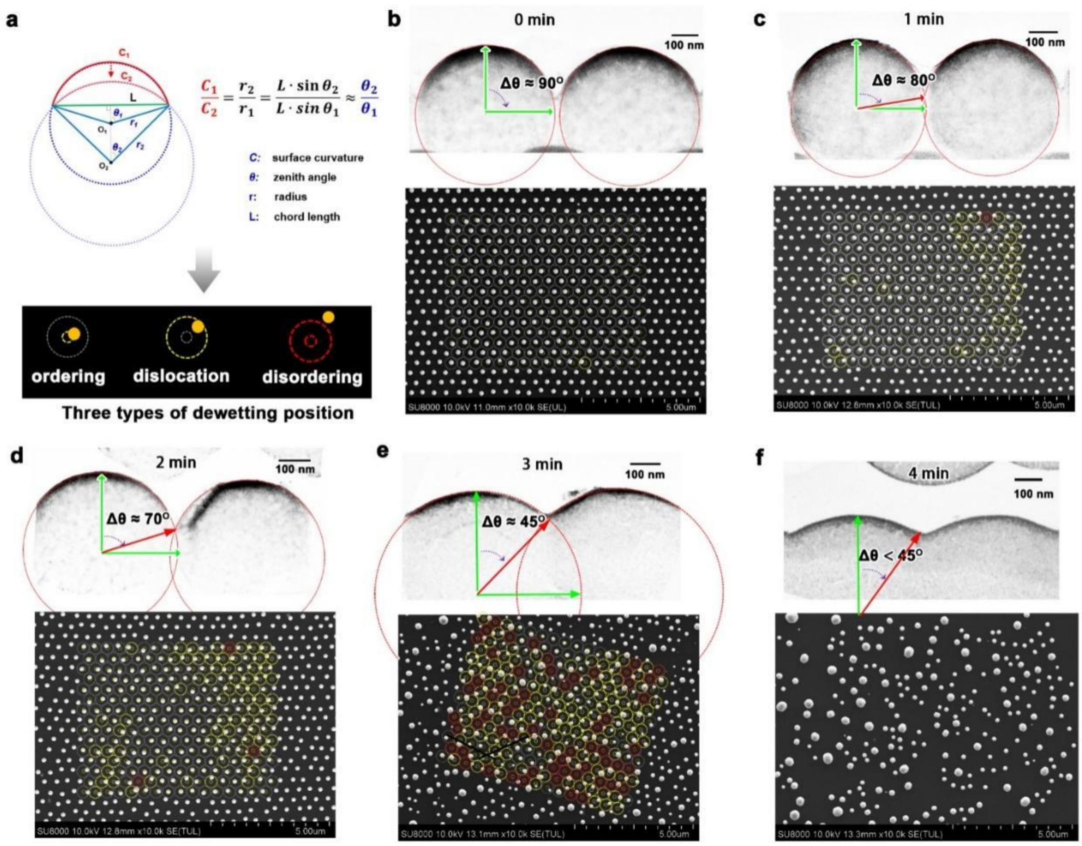

Figure S5. Statistical analysis of the evolution of dewetting position of gold nanoparticles after a tuning of the gold nanoshell film from quasi-continuous to continuous state. (a) Schematic illustration of the cutoff Zenith angle ($\Delta\theta$) of the gold nanoshell along the ΓM radial direction, which can be measured from typical TEM image of two adjacent gold nanoshells in a cross-sectional view. The cutoff Zenith angle is used to describe the continuous extent of the PS beads after melting. These dewetted nanoparticles can be categorized into three types: ordering, dislocation and disordering, based on whether the final formed nanoparticle (yellow circle) was positioned inside, cross or outside of the perimeter of the PS bead template projected on the substrate (dash circle). (b-f) TEM images of gold nanoshells with the cutoff Zenith angle ($\Delta\theta$) varying from 90° (b), 80° (c), 70° (d), 45° (e) and smaller than 45° (f), and their corresponding SEM images of the formed gold nanoparticle arrays under a full dewetting process.

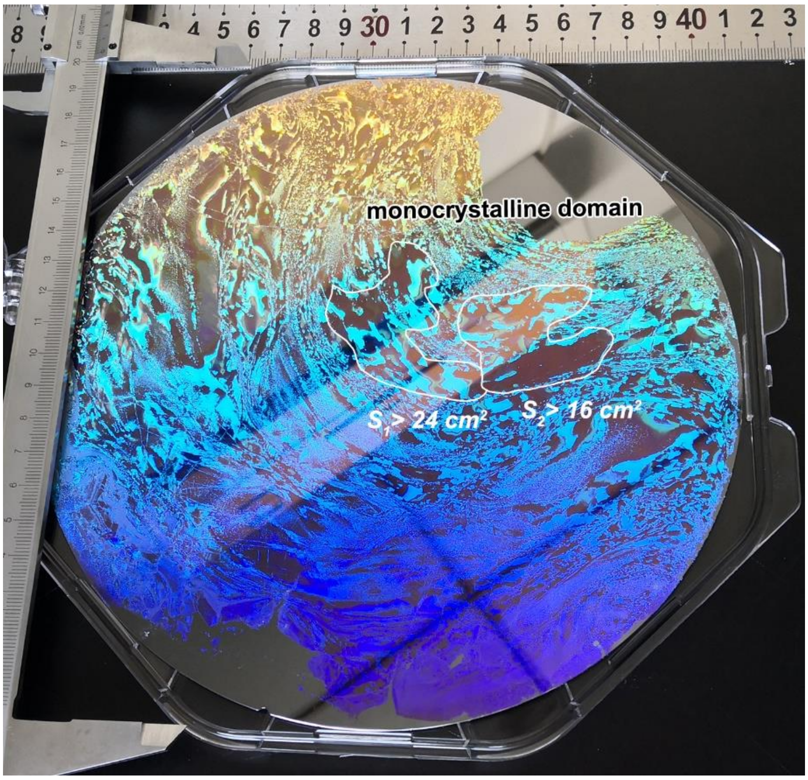

Figure S6. A typical colloidal crystal array assembled on 8-inch silicon substrate based on the air-water interfacial self-assembly technique. The area circled in the white line is two monocrystalline domains with long-range ordering of PS array, where their surface areas are larger than 16 and 24 cm^2, respectively.

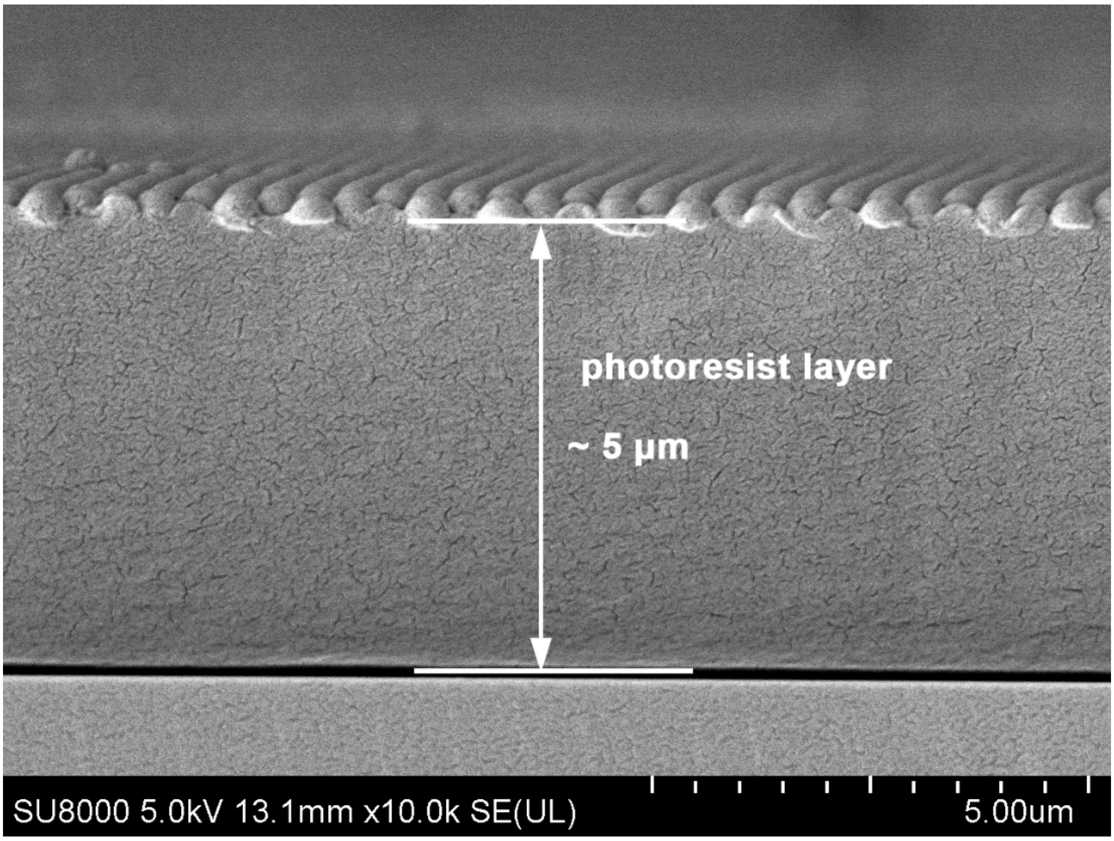

Figure S7. Cross-sectional SEM image of a typical photoresist layer obtained by soft lithography. The photoresist layer is normally about 5 micrometers in thickness.

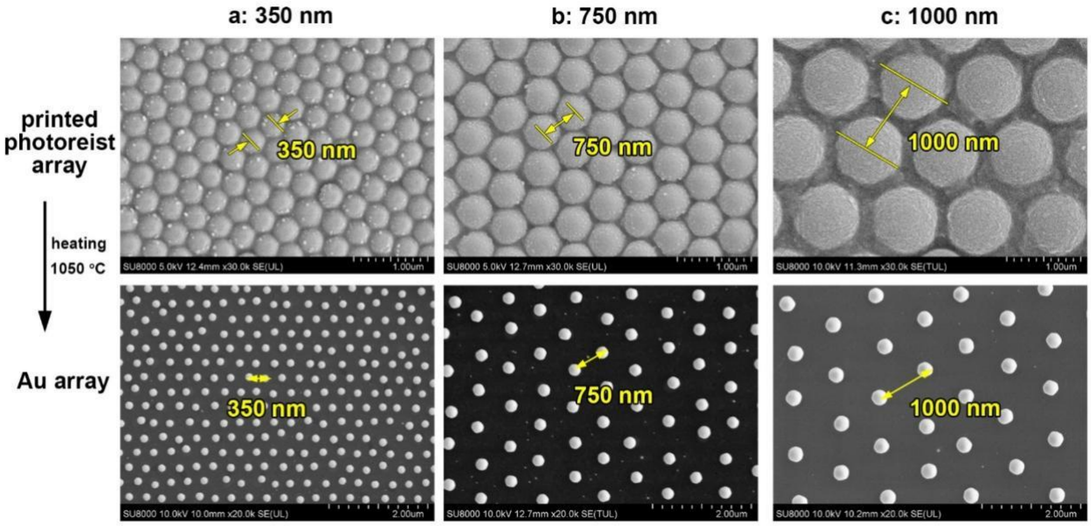

Figure S8. The generality of the soft lithography technique in fabricating gold arrays with different periodicity. Typical SEM images of the printed photoresist arrays and the final-dewetted gold nanoparticle arrays with 350 nm (a), 750 nm (b), and 1000

nm(c) in periodicity.

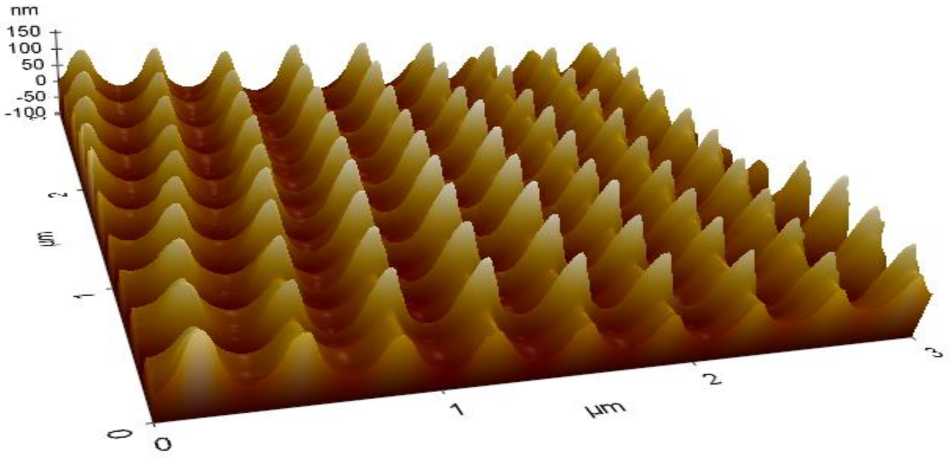

Figure S9. A typical AFM image of the Al_2O_3 template used for soft lithography, which has a predesigned quadrilateral arrangement.

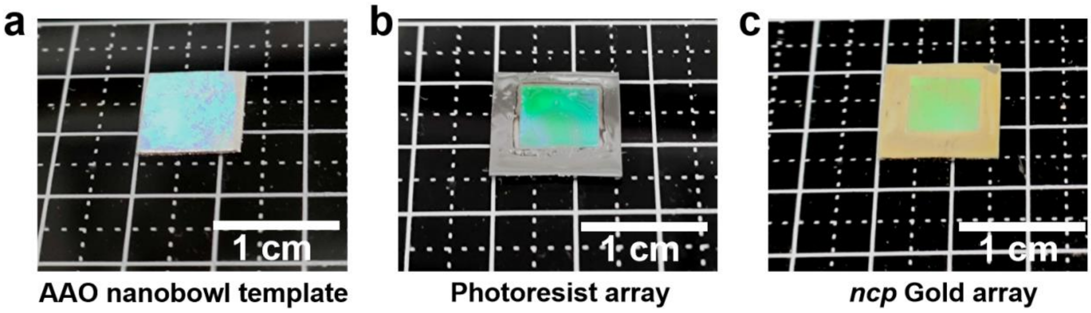

Figure S10. Typical photographs of the AAO template (a), printed photoresist array (b) and dewetted *ncp* gold NPs array (c) in a tetragonal arrangement.

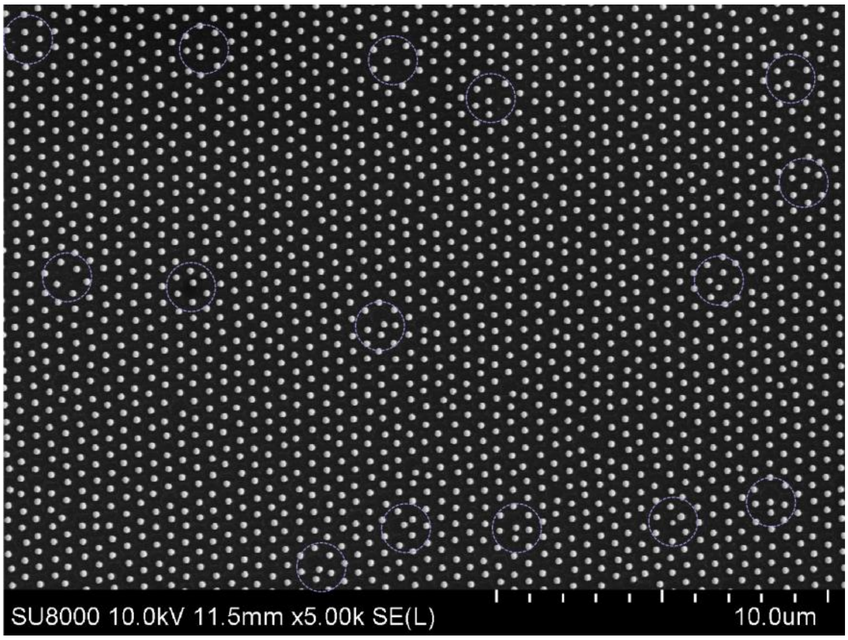

Figure S11. SEM of the defect caused by the deviation of the labeled gold NPs position in the prepared array.

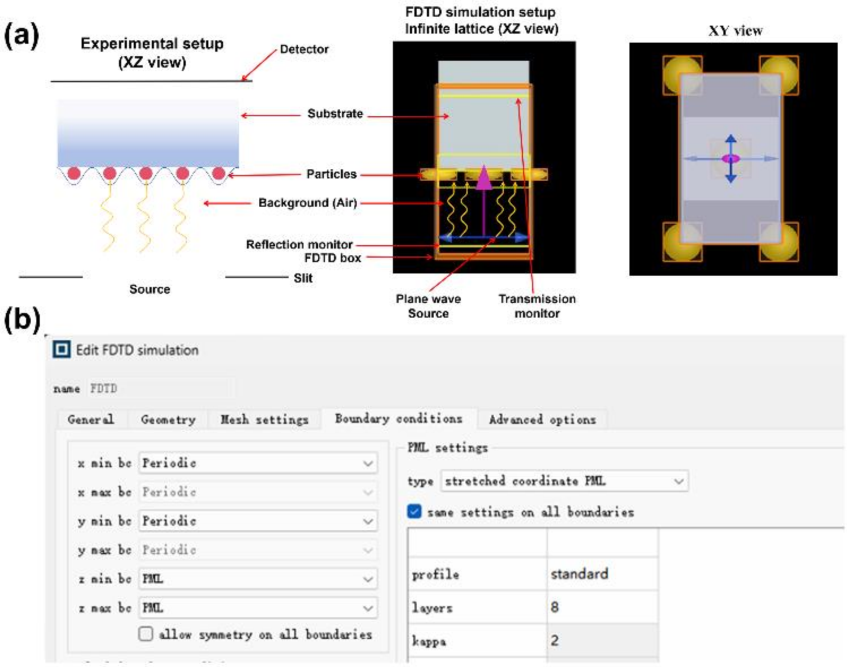

Figure S12. Schematic representation of the experimental transmission measurements and the FDTD simulation conditions for the infinite lattice.

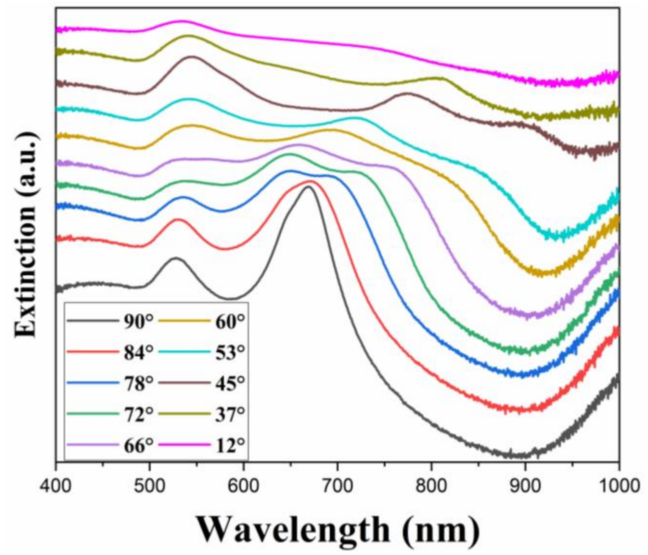

Figure S13. The extinction spectra of *ncp* gold array from angular-dependent measurements.

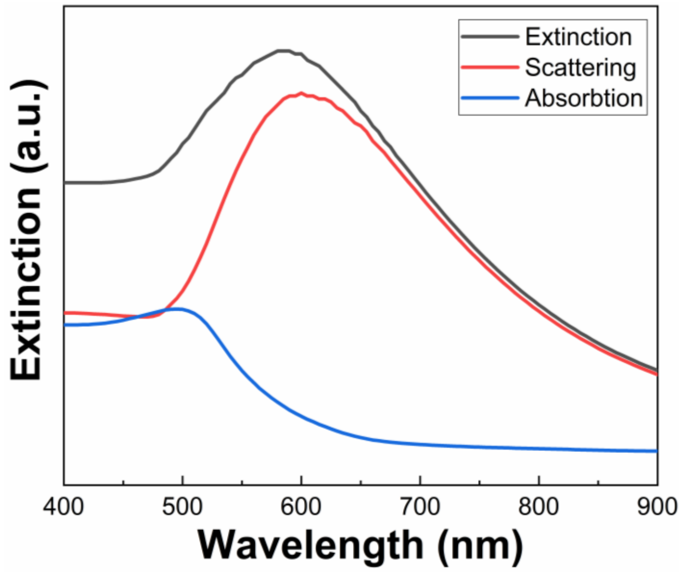

Figure S14. The extinction, absorption and scattering cross sections of a single gold NP. (This tool uses Mie Theory to calculate the optical cross-sections of single-component)

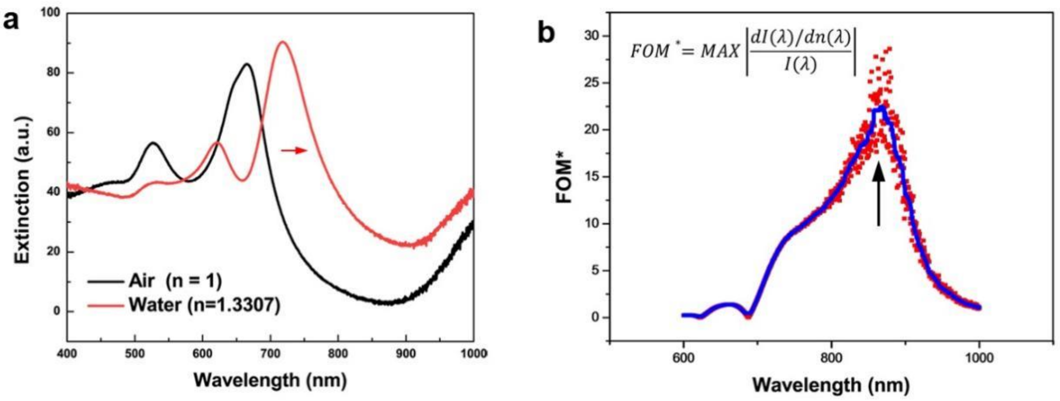

Figure S15. (a) The extinction spectra of gold nanoparticle array relating to the changes of the dielectric environment from air to water. (b) An experimental figure of merit (FOM*) as a function of wavelength based on the spectra results of (a).

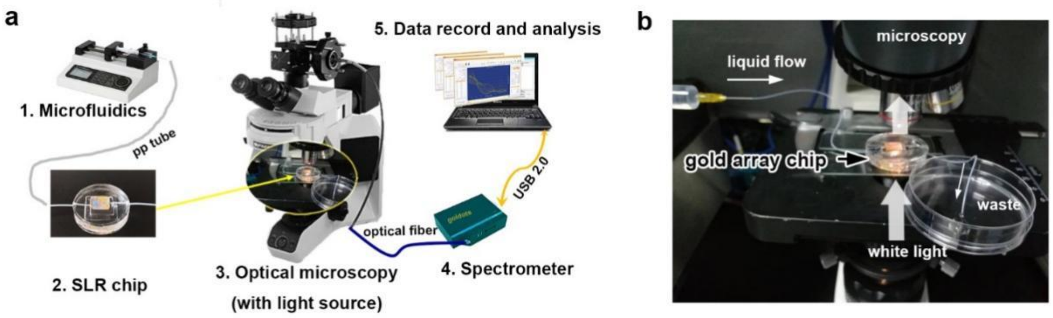

Figure S16. The equipment setup for the molecular-interaction sensing based on a *ncp* gold nanoparticle array. (a) The typical equipment setup containing five basic parts, including the microfluidics, the gold array chip sealed in predesigned PMDS mold, the optical microscopy that provides light source, the spectrometer, and the computer for date record and analysis. (b) A typical photography of the SLR equipment built on our lab.

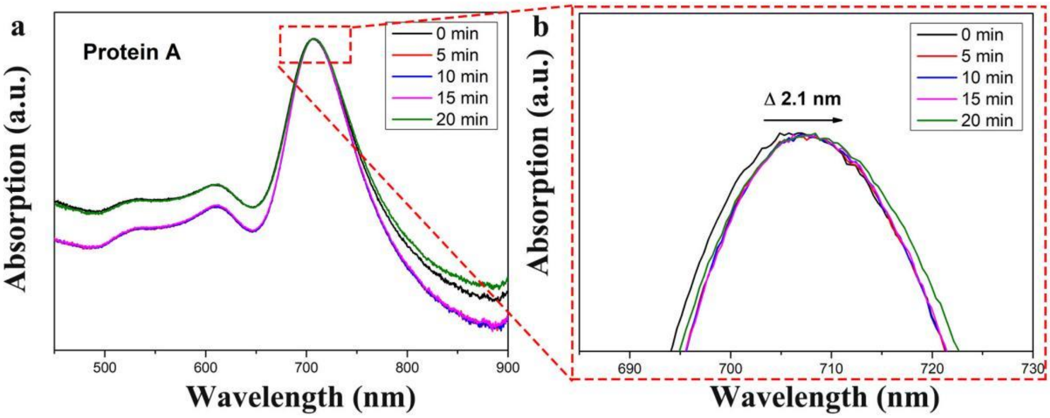

Figure S17. (a) The extinction spectra of the *ncp* gold array after functioned with Protein A molecules (50 μg/mL) working as recognizing element. (b) The corresponding magnification of the SLR peak of (a).

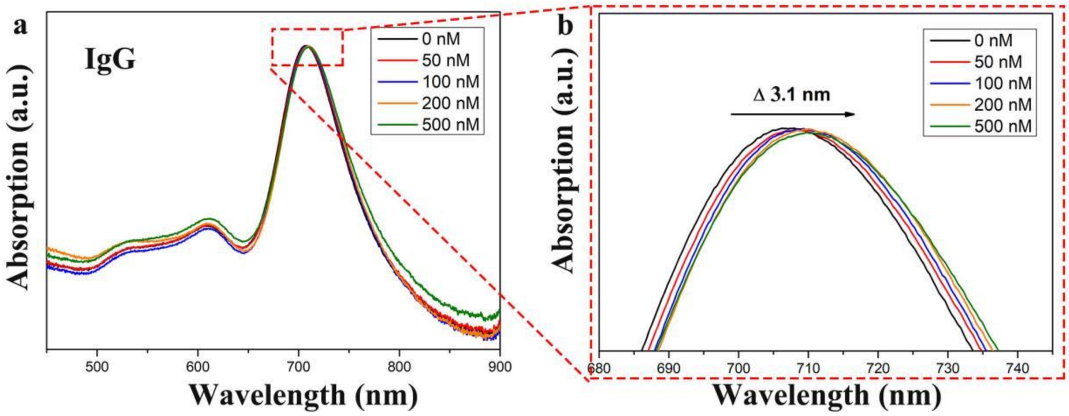

Figure S18. (a) The extinction spectra of the *ncp* gold array in response to the binding of the IgG molecules at different concentrations. (b) The corresponding magnification of the SLR peak in (a).

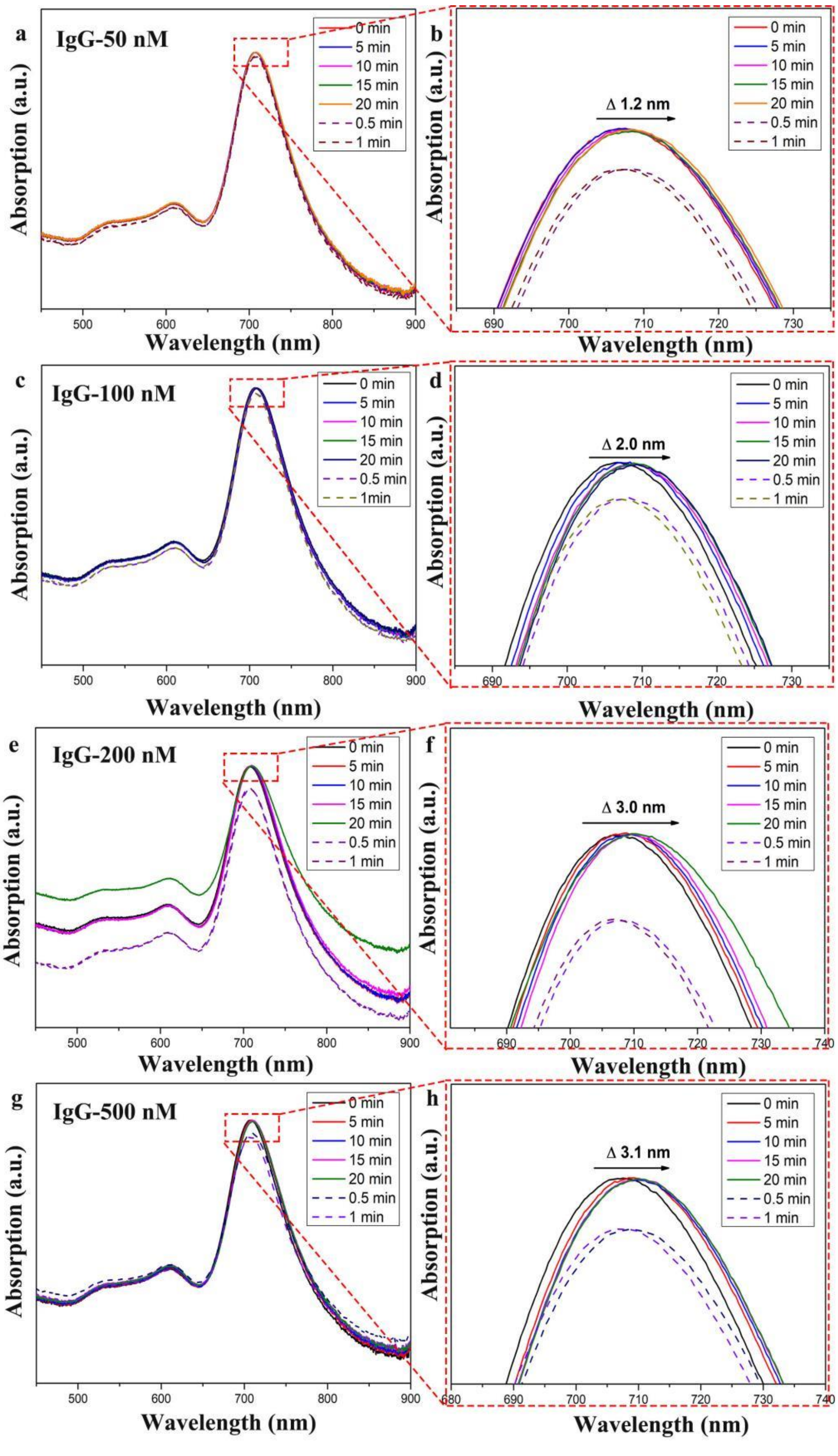

Figure S19. (a-h) The extinction spectra of the *ncp* gold array in response to the binding of IgG molecules with the concentration varying from 50 to 500 nM, notes: the solid line represents the binding process and the dashed line represents the regenerated process.

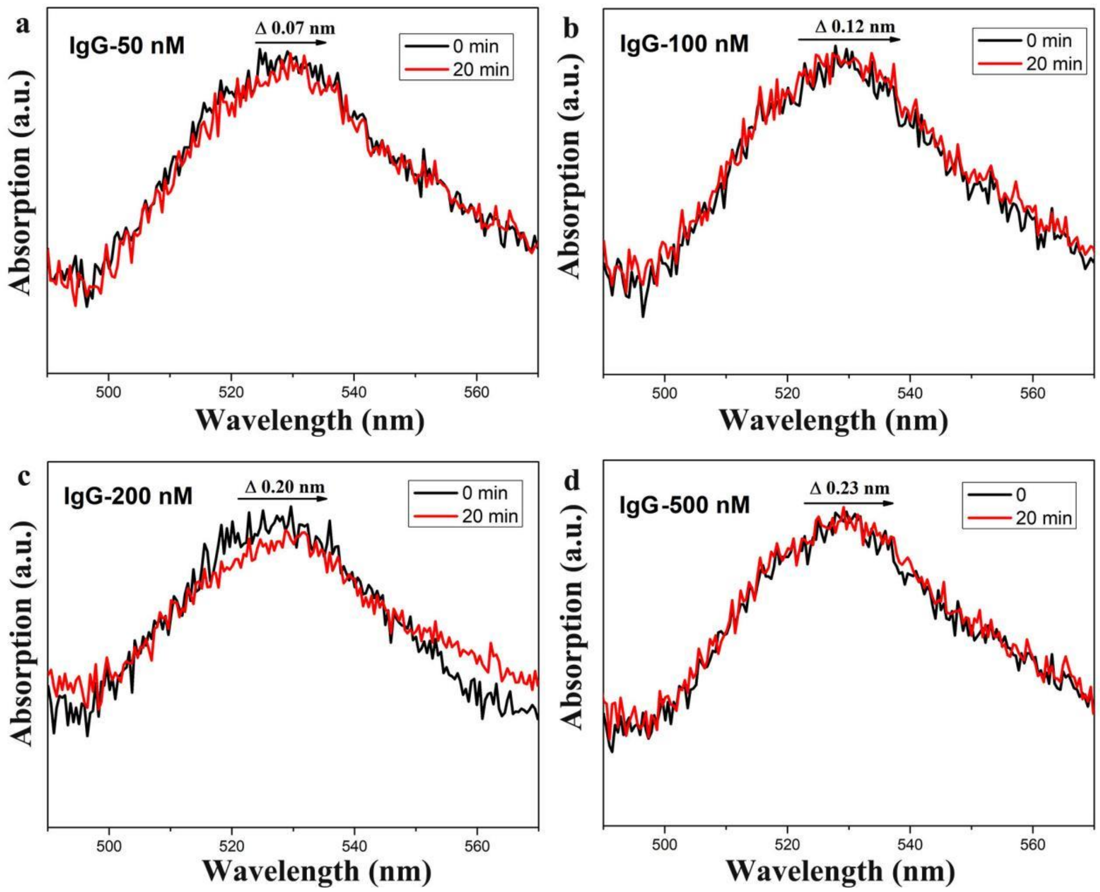

Figure S20. (a-d) The relatively LSPR peak of the gold nanoparticles in array in response to the binding of the IgG molecules with the concentration varying from 50 to 500 Nm.

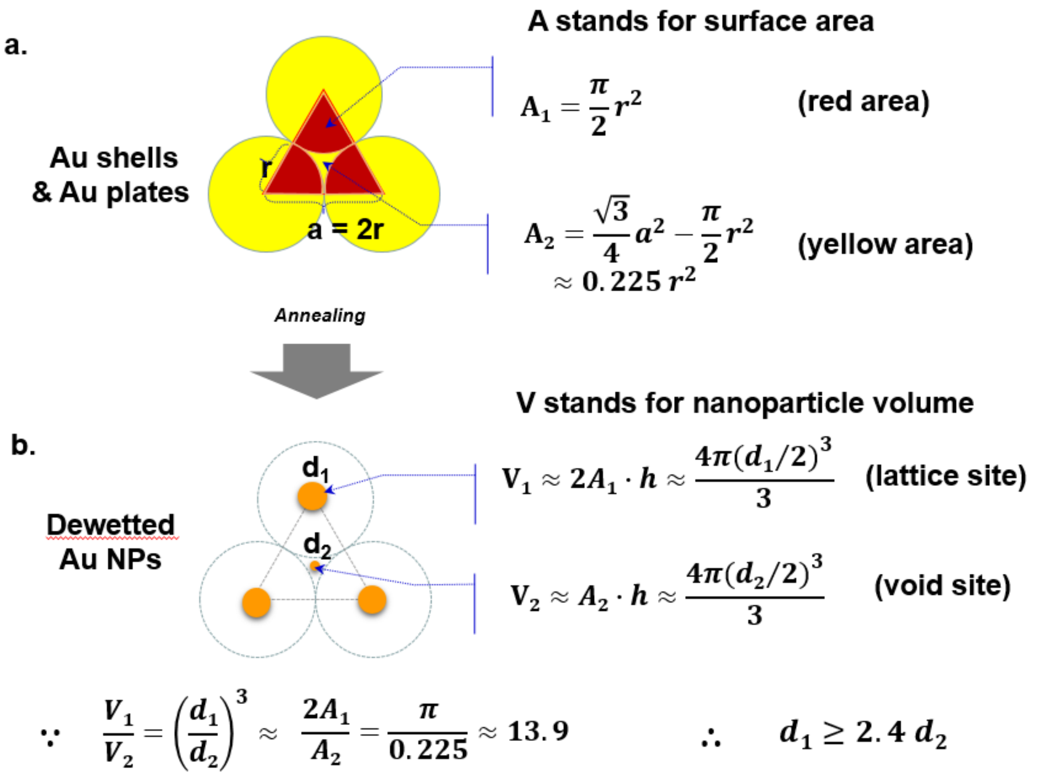

Figure S21. The calculations of the surface areas and the corresponding dewetted nanoparticle volumes at the lattice site and the void site of the colloidal crystal.

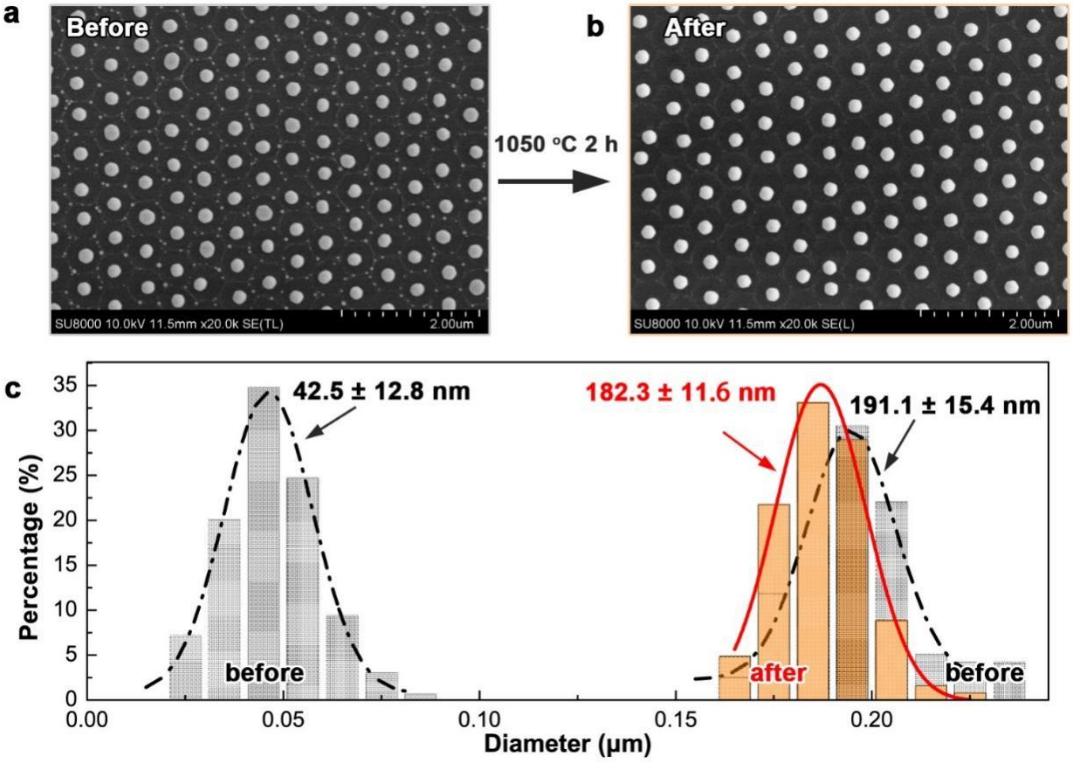

Figure S22. Eliminating the small and random gold nanoparticles by slightly

increasing the annealing temperature. SEM images of a dewetted gold nanoparticle array before (a) and after (b) an additional slight gold evaporation process at 1050 ^{o}C for 2 h. (c) The statistics of the size distribution of the gold nanoparticles according to the SEM images of (a) and (b).

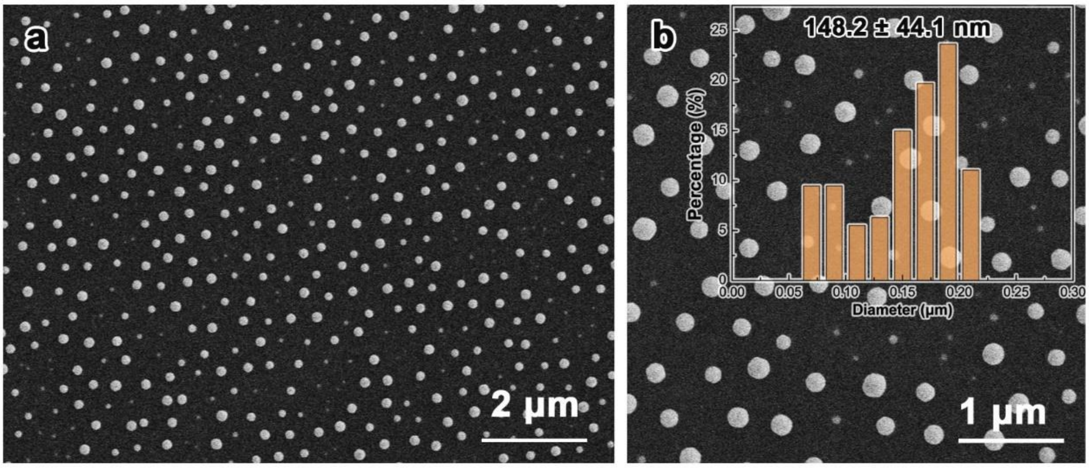

Figure S23 The periodicity of the gold array destructed by a severe gold evaporation at high temperature. (a, b) Typical SEM images of a gold nanoparticle array after an additional annealing process at 1100 ^{o}C for 2 h. Inset in (b) shows the corresponding size distribution of the dewetted gold nanoparticles.

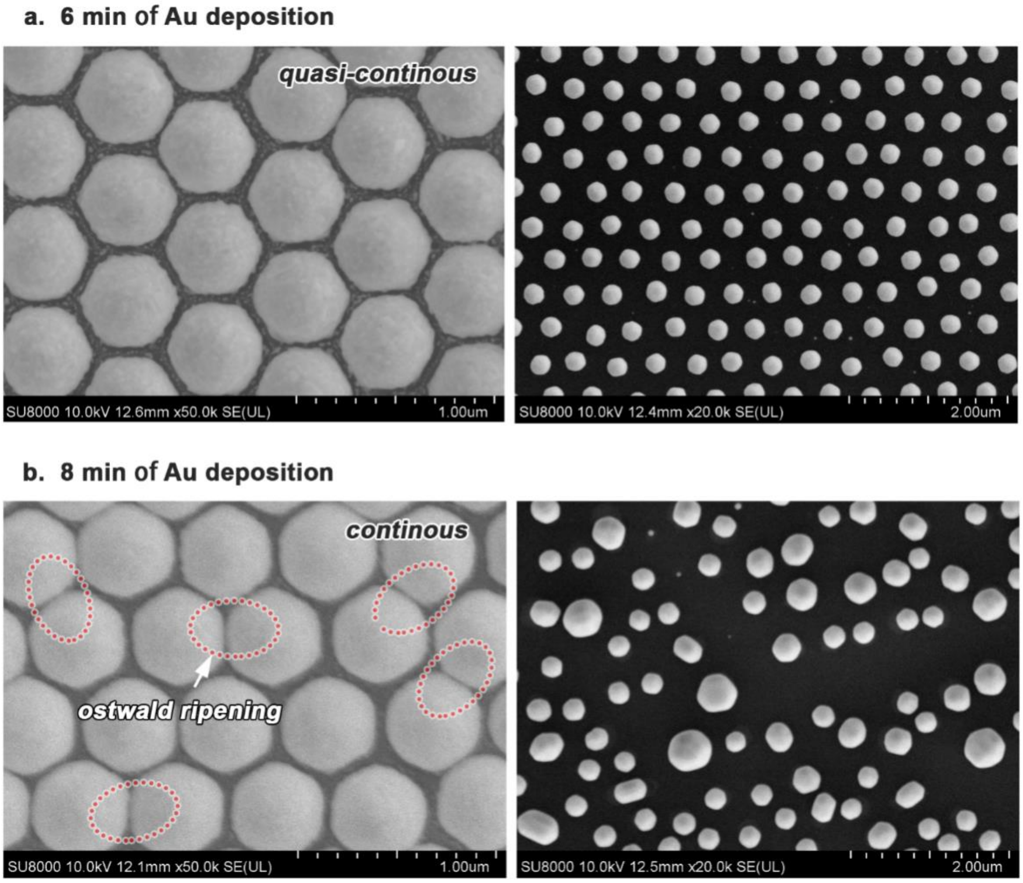

Figure S24. The localized Ostwald ripening occurs after an excessive gold deposition. The SEM images gold nanoshell and gold nanoparticle arrays obtained at 6 min of gold deposition (a), which can still remain the quasi-continuous state of arrangement, while at 8 min of gold deposition (b), it will cause the adjacent gold nanoshell merging into a continuous state, and then leads to a localized Ostwald ripening during the dewetting process.

References

[1] D. Liu, W. Cai, M. Marin, Y. Yin, Y. Li, *ChemNanoMat* **2019**, 5, 1338.

[2] Tao Zhang, Y. Sun, L. Hang, H. Li, G. Liu, X. Zhang, X. Lyu, W. Cai, Y. Li, *ACS Appl. Mater. Interfaces* **2018**, 10, 9792.

[3] C. Xing, S. Zhong, J. Yu, X. Li, A. Cao, D. Men, B. Wu, W. Cai, Y. Li, *J. Mater. Chem. C* **2020**, 8, 3838.

[4] B. Gosselin, M. Retout, R. Dutour, L. Troian-Gautier, R. Bevernaegie, S. Herens, P. Lefevre, O. Denis, G. Bruylants, I. Jabin, *Anal. Chem.* **2022**, 94, 7383.

[5] V. G. Kravets, A. V. Kabashin, W. L. Barnes, A. N. Grigorenko, *Chem. Rev.* **2018**, 118, 5912.

[6] S. Zou, N. Janel, G. C. Schatz, *J. Chem. Phys.* **2004**, 120, 10871.

[7] E. Ponomareva, K. Volk, P. Mulvaney, M. Karg, *Langmuir* **2020**, 36, 13601.

[8] S. Ye, H. Zha, Y. Xia, W. Dong, F. Yang, C. Yi, J. Tao, X. Shen, D. Yang, Z. Nie, *ACS Nano* **2022**, 16, 4609.

[9] J. Guan, R. Li, X. G. Juarez, A. D. Sample, Y. Wang, G. C. Schatz, T. W. Odom, *Adv. Mater.* **2023**, 35, 2103262.
